# Transformation-Aware Molecular Networking for Interpretation of Untargeted LC–HRMS Data

**DOI:** 10.1021/acsmeasuresciau.6c00011

**Published:** 2026-03-26

**Authors:** Elena Ferri, Cristian Caprari, Maria Angela Vandelli, Rossana Cecchi, Patrizia Verri, Cinzia Citti, Giuseppe Cannazza

**Affiliations:** † Department of Life Sciences, 9306 University of Modena and Reggio Emilia, Via Campi 103 41125, Modena, Italy; ‡ Health Innovative Products and Technologies (HIP-TECH) PhD Program, Department of Life Sciences, University of Modena and Reggio Emilia 41125, Modena, Italy; § Clinical and Experimental Medicine (CEM) PhD Program, Nanomedicine, Medicinal and Pharmaceutical Sciences, Department of Life Sciences, 518742 University of Modena and Reggio Emilia 41125, Modena, Italy; ∥ Department of Biomedical, Metabolic and Neural Sciences, Institute of Legal Medicine, University of Modena and Reggio Emilia 41125, Modena, Italy; ⊥ Institute of Nanotechnology-CNR NANOTEC, Campus Ecotekne, Via Monteroni 73100, Lecce, Italy

**Keywords:** transformation-aware molecular networking, untargeted LC–HRMS, metabolite identification, forensic toxicology, biotransformation pathways, new psychoactive substances

## Abstract

The rapid emergence of new psychoactive substances (NPS) and their extensive biotransformation challenge the reliability and interpretability of chemical measurements in forensic toxicology. Targeted analytical workflows offer high selectivity but frequently fail when parent compounds are present at low concentrations or are absent from biological matrices, limiting metabolite coverage and evidential interpretation. In this study, an untargeted LC–HRMS measurement and data-analysis framework was evaluated to improve metabolite annotation and structural contextualization in complex forensic samples. Blood and urine collected from a suspected driving under the influence of drugs (DUID) case were analyzed using high-resolution full-scan and data-dependent MS/MS acquisition as a representative test system. Data were processed in Compound Discoverer using a customized workflow combining spectral library matching (*mzCloud* and *in-house* spectral database built from *HighResNPS.com*, containing over 2400 high-resolution spectra of drugs of abuse and NPS), rule-based metabolite prediction (MetID), and transformation-aware molecular networking. The transformation-aware molecular networking strategy integrates MS/MS spectral similarity with predicted phase I and phase II biotransformations, enabling relational organization of parent compounds, metabolites, and structurally related features. Compared to rule-based metabolite prediction alone, this approach increased the number of metabolite-related features associated with detected xenobiotics and supported reconstruction of chemically consistent metabolic families, including cases in which parent compounds were not observed in the measured sample. Taken together, the results show that transformation-aware molecular networking provides an effective means of organizing and interpreting untargeted LC–HRMS data by linking spectral similarity with biotransformation relationships. This framework supports a more contextual interpretation of metabolite-related features in forensic and toxicological investigations involving NPS and other xenobiotics.

## Graphical abstract

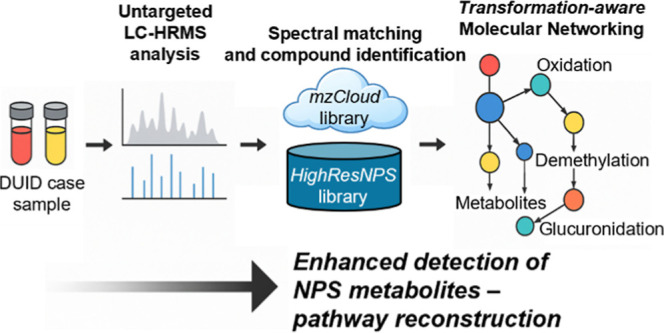

## 1 Introduction

The rapid expansion of the synthetic drug market and the continuous emergence of structurally diverse psychoactive compounds pose significant challenges for reliable chemical measurement and data interpretation in forensic toxicology. In particular, the increasing prevalence of new psychoactive substances (NPS) has exposed fundamental limitations in conventional analytical workflows, which are often optimized for the targeted detection of a limited number of known compounds. According to the 2025 World Drug Report, illicit drug use has continued to rise globally, both in absolute numbers and prevalence, reinforcing the need for analytical strategies capable of addressing chemically evolving targets in complex biological matrices.^1^

From a measurement perspective, NPS present a dual analytical challenge. First, their structural diversity frequently limits detectability by routine immunoassays and targeted liquid chromatography coupled tandem mass spectrometry (LC–MS/MS) methods.^2^ Second, many NPS undergo rapid and extensive biotransformation, resulting in short parent-compound half-lives and the predominance of metabolites in biological samples.^3,4^ Under these conditions, analytical workflows that rely on the direct detection of parent drugs provide incomplete chemical coverage and limited evidential context. Consequently, metabolite-centered strategies are essential for extending detection windows and improving interpretability in toxicological measurements.

Untargeted analytical approaches based on liquid chromatography coupled to high-resolution mass spectrometry (LC–HRMS) have therefore become central to contemporary forensic and toxicological investigations.^5–7^ These techniques enable comprehensive acquisition of chemical features and provide accurate mass and fragmentation information for both known and unknown compounds. However, the analytical value of untargeted LC–HRMS data is ultimately constrained by postacquisition data processing and interpretation. Traditional metabolite annotation strategies—including spectral library matching, mass defect filtering, and neutral loss filtering—have been successfully applied for decades but exhibit important limitations when confronted with large-scale untargeted data sets.^8,9^

A major limitation arises from incomplete spectral coverage in existing reference databases. More than 90% of metabolites listed in widely used resources such as METLIN, HMDB, KEGG, and DrugBank lack well-characterized MS/MS spectra, complicating confident annotation. This limitation is particularly pronounced for drugs of abuse and NPS, for which analytical standards are frequently unavailable and novel analogues are continuously introduced. In addition, the complexity of biological matrices often necessitates extensive manual inspection and validation of candidate features, reducing throughput and introducing subjectivity into the interpretation process.^8,10^

Molecular networking (MN) has emerged as a data-analysis strategy for organizing and interpreting untargeted mass spectrometry data through relational analysis of MS/MS spectra.^2,11^ By representing ions as nodes and connecting them based on spectral similarity, MN enables visualization of structural relationships and supports propagation of structural information from known to unknown features.^9,12^ Both classical molecular networking (CMN) and feature-based molecular networking (FBMN) have been successfully applied in metabolomics and forensic toxicology, including the investigation of NPS-related compounds.^5,13–16^ FBMN improves network accuracy by incorporating chromatographic alignment, isotopic patterns, and relative abundance information.

Despite these advances, conventional MN approaches remain limited in their ability to contextualize metabolites within coherent biotransformation pathways. Spectral similarity alone may fail to associate structurally divergent metabolites or low-abundance transformation products, particularly when parent compounds are absent or obscured by co-occurring substances such as adulterants. As a result, a need remains for data-analysis frameworks that explicitly integrate biochemical transformation logic with spectral similarity to improve metabolite coverage and interpretative context.

In this work, a transformation-aware molecular networking framework was implemented to integrate MS/MS similarity with predicted phase I and phase II biotransformations. Blood and urine samples collected from a suspected driving under the influence of drugs (DUID) case were therefore used as a realistic test system to evaluate how this framework supports reconstruction of metabolic relationships among co-occurring xenobiotics in complex untargeted HRMS data sets.

## 2 Materials and Methods

### 2.1 Chemicals and Materials

LC–MS-grade acetonitrile (ACN) and formic acid (FA) were purchased from Honeywell (Charlotte, NC, USA). Ultrapure water used for LC–HRMS analyses was produced using a Direct-Q 3UV purification system (Merck Millipore, Milan, Italy). LC–MS-grade methanol (MeOH) was obtained from Baker-VWR (Milan, Italy), and ammonium formate (analytical grade) was supplied by Carlo Erba (Milan, Italy). Certified reference standards of cocaine (COC; 1 mg mL^–1^ in ACN), benzoylecgonine (BZE), midazolam (MDZ), and acetaminophen (APAP; all 1 mg mL^–1^ in MeOH) were obtained from Sigma-Aldrich Merck (Darmstadt, Germany). Phree Phospholipid Removal cartridges (1 mL) were purchased from Phenomenex (Torrance, CA, USA) and used to remove proteins and phospholipids from blood samples prior to LC–HRMS analysis.

Peripheral blood and urine samples were provided by the Institute of Legal Medicine, University of Modena and Reggio Emilia, and were used as representative biological matrices for evaluation of the analytical workflow.

### 2.2 Sample Preparation

The biological samples were collected within routine forensic casework at the Department of Legal Medicine of Modena. The present study involved retrospective analysis of fully anonymized samples, and no additional interventions or sample collection were performed for research purposes. The study was conducted in accordance with institutional procedures and national regulations governing forensic investigations.

Aliquots of 200 μL of blood or urine were mixed with 1 mL of a 1 mM ammonium formate solution in ACN/MeOH (70:30, v/v) containing 0.1% FA, following a protocol previously validated for post-mortem and forensic toxicological analyses.^17^ Samples were vortex-mixed for 2 min and centrifuged at 5300 rpm for 8 min. Supernatants were purified using Phree phospholipid removal cartridges to reduce matrix-related ion suppression effects.

Extracts were evaporated to dryness under a gentle nitrogen stream at 40 °C for approximately 6.5 h, and reconstituted in 100 μL of ACN, followed by dilution with 100 μL of ACN/MeOH (50:50, v/v) containing 0.1% FA. After sonication at 40 °C for 15 min, samples were transferred to LC vials, and 5 μL aliquots were injected for LC–HRMS analysis. Reference standard solutions (10 μg mL^–1^ in ACN or MeOH, depending on solubility) were prepared and analyzed under identical conditions to support compound confirmation.

Sample handling and LC–HRMS analyses were conducted following standard laboratory safety procedures for handling biological specimens and organic solvents. No unexpected hazards were identified.

### 2.3 LC–HRMS Analysis

Analyses were performed using a Thermo Fisher Scientific LC–HRMS system (Waltham, MA, USA) consisting of an Ultimate 3000 ultra-high performance liquid chromatograph coupled to a Q-Exactive quadrupole-Orbitrap high-resolution mass spectrometer equipped with a heated electrospray ionization (HESI) source. The UHPLC system included a binary pump, a vacuum degasser, an autosampler thermostated at 7 °C, and a column compartment maintained at 25 °C. The mass spectrometer was operated in positive ionization mode for all acquisitions.

Chromatographic separation was achieved on a Poroshell 120 EC–C18 column (100 × 3.0 mm i.d., 2.7 μm) with a guard column (5 × 3 mm, 2.7 μm) (Agilent Technologies, Milan, Italy). The mobile phases were (A) water and (B) acetonitrile, both containing 0.1% formic acid. A linear gradient was applied from 5% to 95% B over 2–20 min, held at 95% B for 5 min, and returned to 5% B at 25.1 min, followed by 5 min of re-equilibration, for a total run time of 30 min.

The Orbitrap analyzer operated in full-scan (FS) mode over an *m*/*z* range of 50–750. Data-dependent acquisition (DDA) was subsequently performed to obtain MS/MS spectra of parent compounds and major metabolites. HESI source parameters were: capillary temperature 320 °C, vaporizer temperature 300 °C, spray voltage 3.8 kV, sheath gas 55 au, auxiliary gas 30 au, and S-lens RF level 55 au The Orbitrap was calibrated daily and a lock mass list was applied for improved mass accuracy.

Data acquisition was managed using Xcalibur 3.0 (Thermo Fisher Scientific, San Jose, CA, USA). In full-scan mode, resolution was set to 140,000 (fwhm) with an AGC target of 3 × 10^6^ and maximum injection time (IT) of 500 ms. For DDA MS/MS, the AGC target was 5 × 10^5^, minimum intensity threshold 1 × 10^3^, resolution 17,500, IT 100 ms, and isolation window 0.4 *m*/*z*. Normalized collision energy (NCE) was 20 au, with a loop count of 2 and dynamic exclusion of 5 s. Data was inspected and visualized using FreeStyle 1.8 (Thermo Fisher Scientific).

### 2.4 Untargeted Data Processing

Untargeted data processing was performed using Compound Discoverer (CD) version 3.4 (Thermo Fisher Scientific). A customized workflow derived from the “Forensics Unknown ID with Compound Class Scoring and Database Searches” template was implemented to ensure consistent feature detection, alignment, and annotation across samples (Figure S1). Only positive-ion data were considered.

Feature detection employed a mass tolerance of 5 ppm, with retention time alignment performed using the adaptive curve model (maximum shift 2 min). Features were grouped across samples using a retention time tolerance of 0.2 min, and adduct assignment prioritized [M + H]^+^ and [M + Na]^+^ species. Minimum intensity (1 × 10^4^), signal-to-noise ratio (≥1.5), and peak rating (≥3) thresholds were applied to improve analytical reliability. Elemental compositions were predicted using defined constraints (C_1–90_ H_1–190_ Br_0–3_ Cl_0–4_ K_0–4_ N_0–10_ O_0–18_ P_0–3_ S_0–5_) and background features present in procedural blanks were excluded.

Spectral annotation was performed using *mzCloud* and a custom *mzVault* library containing over 2400 high-resolution MS/MS spectra of drugs of abuse and NPS compiled from *HighResNPS.com*. This combined library strategy was used to maximize spectral coverage while maintaining consistent scoring criteria.

### 2.5 Metabolite Prediction Using MetID

Rule-based metabolite prediction was performed using the MetID module implemented in CD (Figure S2). Expected metabolites were generated for five parent compounds COC, MDZ, dextromethorphan (DXM), levamisole (LEV), and APAP by applying common phase I and phase II biotransformations (Table S1), with a maximum of three sequential transformation steps. Only [M + H]^+^ species were considered. Predicted metabolites were matched to experimental full-scan^1^ features using a 5 ppm mass tolerance and a retention time tolerance of 0.2 min, followed by grouping and annotation through spectral library searches and FISh fragment scoring.

### 2.6 Transformation-Aware Molecular Networking

To integrate relational information into untargeted data interpretation, transformation-aware molecular networking was applied using the “Generate Molecular Networking” node in CD. Network connections were established based on combined criteria of MS/MS spectral similarity and predicted biotransformation relationships. Spectral similarity was evaluated using full MS^n^ tree information with a signal-to-noise threshold ≥3 and a fragment mass tolerance of 2.5 mmu.

A curated set of biotransformation rules relevant to xenobiotic metabolism was applied consistently across the data set (Table S1). The algorithm generated and evaluated all feasible transformation pathways, selected the shortest path where multiple routes converged, and assigned zero-length edges to chromatographically resolved isomers. Validated links were exported to construct the final molecular networks used for interpretation. This approach enables convergence of rule-based metabolite prediction and similarity-driven network analysis by associating predicted transformation products with experimentally observed untargeted features within a unified relational framework.

Importantly, edge formation was not based solely on mass differences. Connectivity required concurrent support from MS/MS spectral similarity, curated biotransformation plausibility, and relational consistency within shortest-path logic. Transformation rules were restricted to physiologically plausible phase I and phase II xenobiotic metabolic reactions (Table S1), and generic or chemically arbitrary mass differences were not considered. Candidate metabolites derived from network connectivity were subsequently evaluated at the compound level, including inspection of fragmentation coherence and, where applicable, comparison with reference standards. These combined constraints enhanced edge specificity and reduced the likelihood of false positive network-derived associations.

### 2.7 Seed-Based Network Analysis

Seed and class compound analysis was performed to facilitate targeted exploration of metabolite families within the molecular network. In this study, the “Seed Compound” functionality was employed to introduce reference nodes based on diagnostic fragment ions derived from representative xenobiotics and their known analogues, using curated high-resolution MS/MS spectra. A custom “Class Compound” library was constructed for this purpose. Details of the diagnostic fragments and compound-specific libraries are provided in the Supporting Information (Tables S2–S6).

These seed compounds guided network propagation by enabling identification of structurally related features and reconstruction of phase I and phase II metabolic relationships based on combined spectral similarity and biotransformation connectivity.

The combined scoring logic underlying transformation-aware network connectivity is schematically illustrated in Figure 1.

**Figure 1:**
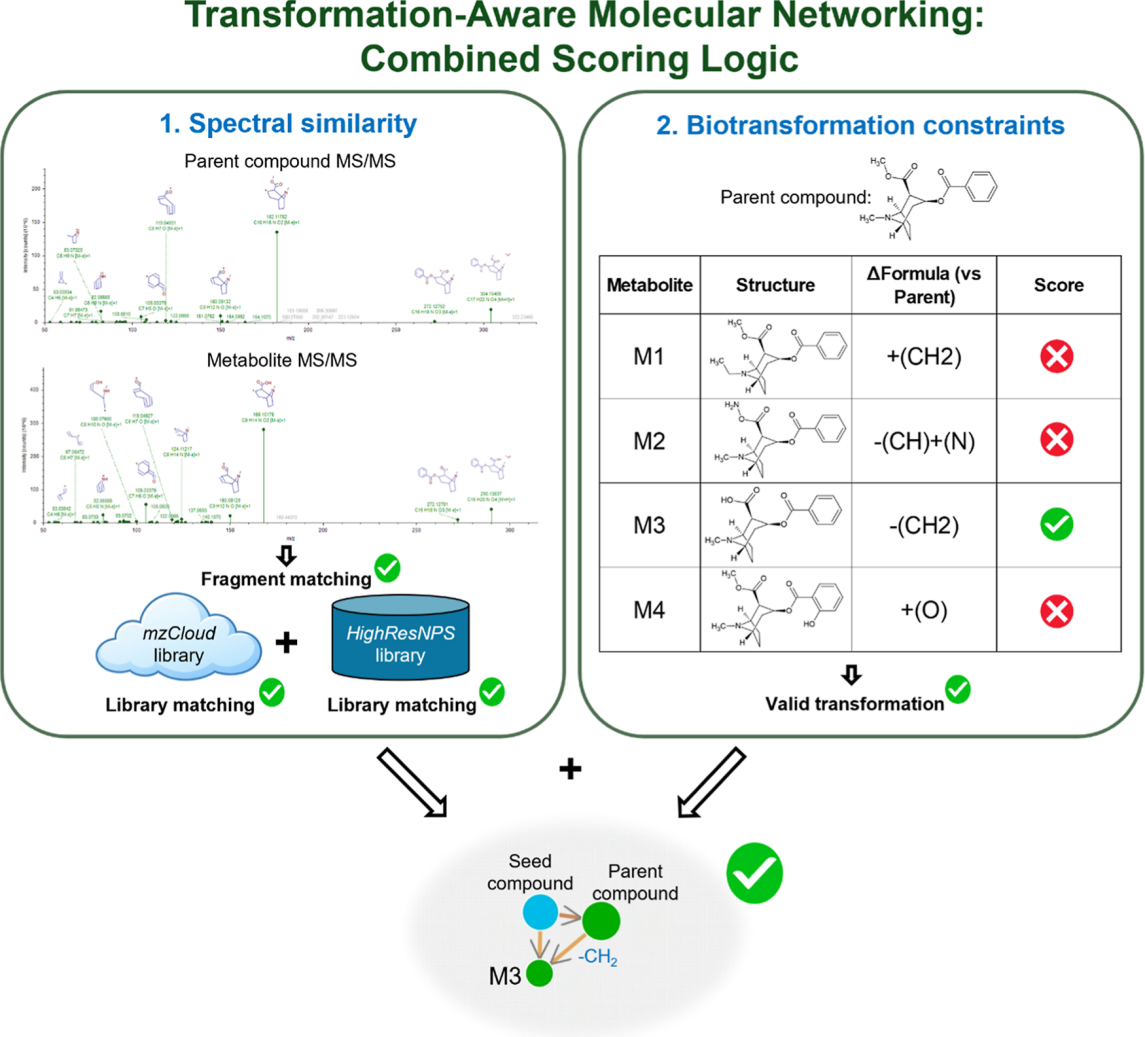
Transformation-aware molecular networking: combined scoring logic integrating MS/MS spectral similarity and biotransformation constraints.

### 2.8 Evaluation of Analytical Artifacts

To minimize the risk of in-source fragmentation (ISF) artifacts being misinterpreted as independent metabolites, chromatographic and spectral criteria were incorporated into the data evaluation workflow. Candidate features were screened for chromatographic independence relative to their proposed parent compounds; no reported metabolite coeluted with its corresponding parent. Because in-source fragments coelute with their precursors, the absence of coelution supports interpretation as distinct chromatographic entities rather than fragment-derived signals.

Chromatographic peak quality was ensured by applying a peak rating threshold (≥3), as described in Section 2.4. MS/MS fragmentation patterns of putative metabolites were compared with those of their parent compounds to verify structural coherence and exclude simple precursor–fragment relationships. These combined criteria reduced the likelihood of fragment-derived artifacts contributing to network connectivity.

The potential impact of electrospray-induced microdroplet chemistry was also considered. Although electrospray-driven reactions have been reported under specific experimental conditions,^18^ analyses were performed using conventional HESI parameters (Section 2.3) routinely applied in forensic toxicology workflows. No evidence of unstable or injection-dependent reaction products was observed. Detected metabolites exhibited coherent distribution patterns across matrices and followed plausible phase I and phase II transformation pathways, reducing the likelihood of artifactual connectivity within the transformation-aware network.

### 2.9 Identification Confidence Assignment

Identification confidence was assigned according to the criteria proposed by Schymanski et al.,^19^ which classify compound annotations from Level 1 (confirmed structure) to Level 5 (exact mass only). Confirmed identifications (Level 1) were assigned to compounds validated using certified analytical standards based on concordant retention time, accurate mass, and MS/MS fragmentation spectra acquired under identical LC–HRMS conditions.

For compounds lacking reference standards, identification confidence was reported as Level 2 or Level 3 depending on the available evidence. Level 2a (probable structure—library) was assigned when experimental MS/MS spectra showed a high-quality and unambiguous match to curated spectral libraries (*mzCloud*, *HighResNPS*), based on consistent precursor mass accuracy, agreement of major fragment ions and their relative intensities with reference spectra, and the absence of conflicting candidate structures. Level 2b (probable structure—diagnostic) was assigned when characteristic fragmentation patterns, biotransformation consistency, and molecular network connectivity collectively supported a single structural assignment in the absence of a reference library spectrum.

Annotations were assigned Level 3 (tentative structure) when positional isomerism could not be resolved (e.g., mono- or dihydroxylated metabolites) or when multiple structural candidates remained plausible based on the available spectral and contextual information. Except for Level 1 identifications confirmed using analytical reference standards, all reported annotations should be considered putative and interpreted accordingly.

For transparency and independent evaluation of structural assignments, the experimental MS/MS fragmentation spectra of all reported metabolites are provided in the Supporting Information as Figures S3–S15 (blood metabolites) and Figures S16–S48 (urine metabolites), including annotated fragment ions and putative structural assignments where applicable.

## 3 Results

### 3.1 Untargeted LC–HRMS Analysis of Blood and Urine Samples

Conventional forensic toxicology workflows typically rely on targeted confirmation guided by preliminary immunoassay screening, which restricts subsequent analysis to a predefined set of compounds. In the present case, immunochemical testing indicated only COC, thereby directing confirmatory analysis exclusively toward this analyte. To overcome the inherent limitations of this targeted strategy and to increase chemical measurement coverage, an untargeted LC–HRMS approach was applied. Blood and urine samples collected from a suspected driving under the influence of drugs (DUID) case were analyzed under positive ESI conditions using full-scan and data-dependent MS/MS acquisition (Section 2.3), and data were processed using the workflow described in Section 2.4. Compound annotation was performed through MS/MS spectral matching against the public *mzCloud* library and a custom *mzVault* database compiled from *HighResNPS.com*, comprising over 2400 high-resolution spectra of drugs of abuse and new psychoactive substances.

Untargeted analysis of the blood sample resulted in the annotation of 2721 features, including xenobiotics such as COC, DXM, and MDZ. COC is among the most widely abused illicit psychoactive substances,^20^ DXM is an opioid-derived antitussive subject to recreational misuse,^21^ and MDZ is a benzodiazepine sedative occasionally encountered in polydrug use scenarios.^22^ Analysis of the urine sample yielded a substantially larger number of annotated features (9907), consistent with the higher abundance and diversity of metabolites typically excreted in this matrix.^23^

In addition to COC, DXM, and MDZ, LEV and APAP were also detected in urine. LEV is an anthelmintic compound frequently reported as an adulterant in illicit cocaine formulations,^24^ while APAP is a widely used antipyretic and analgesic that is also commonly encountered as a cocaine cutting agent.^25,26^ The co-occurrence of these compounds illustrates the broader chemical coverage achieved by the untargeted LC–HRMS workflow across biological matrices and highlights its ability to capture both psychoactive substances and formulation-related components.

COC, MDZ, and APAP were confirmed using certified analytical standards analyzed under identical LC–HRMS conditions, whereas DXM and LEV were supported by high-quality spectral library matches. Notably, DXM, MDZ, LEV, and APAP would not have been captured under a conventional targeted confirmation strategy guided solely by immunoassay screening. This observation highlights a key measurement limitation of targeted workflows, namely their inability to recover therapeutics, adulterants, and secondary psychoactive substances that may contribute to the overall chemical and interpretative context of a sample.

The pronounced difference in the number of annotated features between urine and blood further reflects matrix-dependent metabolic complexity and underscores the importance of untargeted acquisition strategies for comprehensive characterization of xenobiotics and their transformation products. Overall, these results demonstrate how untargeted LC–HRMS acquisition expands the analytical scope beyond predefined target lists, providing a more informative basis for subsequent metabolite-focused and network-based data interpretation.

### 3.2 Rule-Based Metabolite Prediction (MetID)

Following untargeted LC–HRMS acquisition, the data set was reprocessed using the MetID module (Section 2.5) to relate measured features to plausible phase I and phase II biotransformations. MetID was applied uniformly to all five parent compounds considered in this study—COC, DXM, MDZ, LEV, and APAP—in both biological matrices. This uniform application was adopted to avoid excluding metabolites potentially present even when the corresponding parent compound was not observed. For downstream reliability, only features associated with MS/MS spectra and a peak rating greater than 4 were retained; for urine samples, an additional peak-area threshold (>1 × 10^6^) was applied to prioritize analytically robust signals.

In blood, seven metabolites met these criteria. Six were associated with COC biotransformation, including BZE, norbenzoylecgonine (NBZE), hydroxybenzoylecgonine (OH-BZE; two chromatographically resolved isomers), hydroxynorbenzoylecgonine (OH-NBZE; one isomer), and hydroxycocaine (OH–COC; one isomer). For DXM, dextrorphan (DXO), arising from *O*-demethylation, was detected. Although MDZ was present in blood, none of its phase I or phase II metabolites met the applied criteria, and no metabolites related to LEV or APAP were observed. BZE was confirmed using a certified analytical standard, whereas the remaining COC-related metabolites and DXO were supported by spectral library matches and diagnostic MS/MS features, corresponding to identification confidence Levels 2–3. All annotated blood metabolites and their proposed biotransformations are reported in Table S7.

In urine, application of the same criteria resulted in the identification of 20 metabolites attributable to the investigated parent compounds. Twelve metabolites were associated with COC, including BZE, NBZE, OH-BZE (three positional isomers), OH-NBZE (one isomer), norcocaine (NCOC), OH–COC (three positional isomers), dihydroxycocaine (diOH–COC), and cinnamoylecgonine (CEC). Five DXM metabolites were detected, comprising its principal phase I products—DXO (*O*-demethylation), 3-methoxymorphinan (*N*-demethylation), and 3-hydroxymorphinan (3-OH-morphinan) (combined N/O-demethylation)—together with two phase II conjugates, dextrorphan-*O*-glucuronide (DXO-*O*-Glu) and 3-hydroxymorphinan-*O*-glucuronide (3-OH-morphinan-*O*-Glu). MDZ metabolism was supported by the detection of hydroxymidazolam-*O*-glucuronide (OH-MDZ-*O*-Glu), while APAP metabolism was confirmed through acetaminophen-*O*-glucuronide (APAP-*O*-glucuronide) and acetaminophen sulfate (APAP sulfate). No LEV metabolites met the applied criteria. Urine metabolite annotations were supported by spectral library matches and diagnostic MS/MS evidence, with identification confidence assigned as Level 2 or Level 3. A complete overview of annotated urine metabolites and associated transformations is provided in Table S7.

Overall, MetID enabled hypothesis-driven association of measured LC–HRMS features with biologically plausible biotransformation products, capturing major oxidative metabolites of COC and primary phase I metabolism of DXM in blood, as well as a broader range of phase I and phase II metabolites in urine. The contrast between the limited metabolic signature observed in blood and the more extensive metabolite profile detected in urine reflects matrix-dependent differences in metabolite abundance and distribution. While MetID effectively delineated core metabolic routes for the investigated compounds, its rule-based nature limits recovery of metabolites that deviate from predefined transformation patterns. For this reason, transformation-aware molecular networking was subsequently applied to provide complementary, data-driven relational interpretation and to expand metabolite annotation beyond rule-based predictions.

### 3.3 Transformation-Aware Molecular Networking

Transformation-aware molecular networking was applied to integrate MS/MS spectral similarity with predicted biotransformation relationships and to support relational interpretation of untargeted LC–HRMS data (Section 2.6). Global molecular networks generated for blood and urine are shown in Figures 2A and 3A, respectively. At the global level, these networks reflect the expected chemical complexity of biological samples and provide the analytical background against which xenobiotic-related features and their transformation products were evaluated.

**Figure 2:**
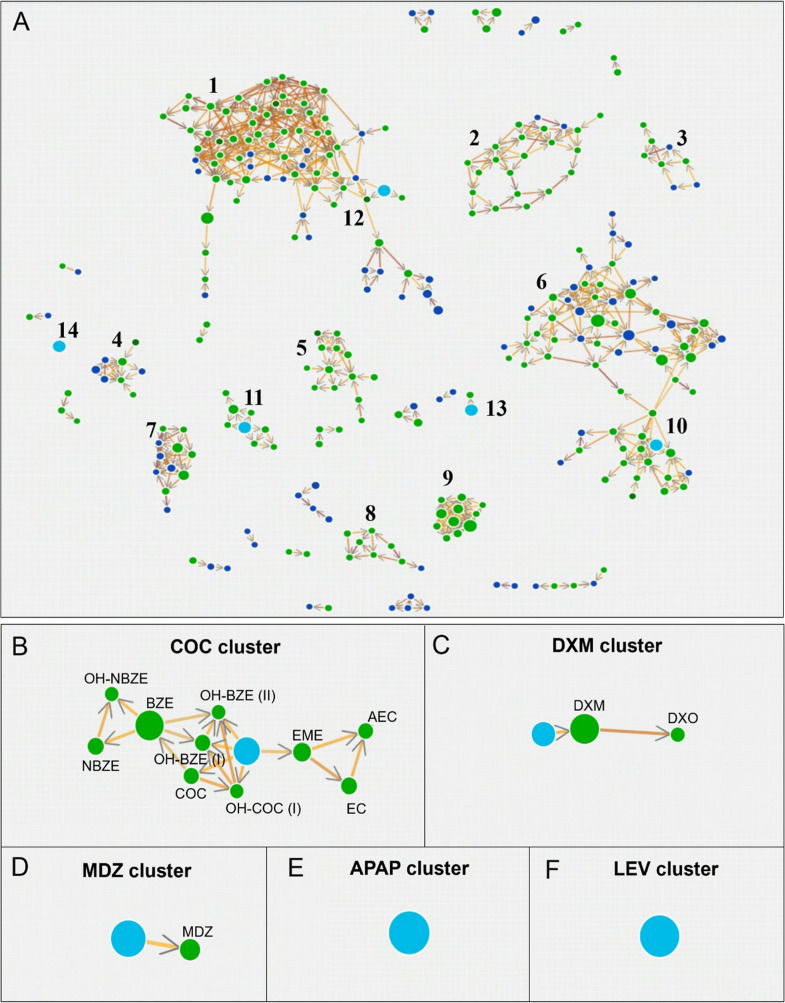
Transformation-aware molecular network of the blood data set. (A) Global network view. The network comprises multiple clusters corresponding to endogenous metabolites and background biological chemistry, including long-chain fatty acid derivatives (Cluster 1), nitrogen- and oxygen-rich high-mass species (Cluster 2), lipid–peptide hybrid structures (Cluster 3), small polar metabolites (Cluster 4), amino acids (Cluster 5), medium-polarity compounds (Cluster 6), glycosidic derivatives (Cluster 7), sterol-related components such as cholecalciferol, vitamin D_3_, and cholesterol (Cluster 9), and small zwitterionic metabolites (Cluster 10). (B–F) Seed-based subnetworks for COC, DXM, MDZ, APAP, and LEV, showing each seed node and, where present, the associated parent compound and connected metabolites. Abbreviations: AEC, anhydroecgonine; APAP, acetaminophen (paracetamol); BZE, benzoylecgonine; COC, cocaine; DXM, dextromethorphan; DXO, dextrorphan; EC, ecgonine; EME, ecgonine methyl ester; LEV, levamisole; MDZ, midazolam; NBZE, norbenzoylecgonine; OH-BZE (I), hydroxybenzoylecgonine (isomer I); OH-BZE (II), hydroxybenzoylecgonine (isomer II); OH–COC (I), hydroxycocaine (isomer I); OH-NBZE, hydroxynorbenzoylecgonine.

**Figure 3:**
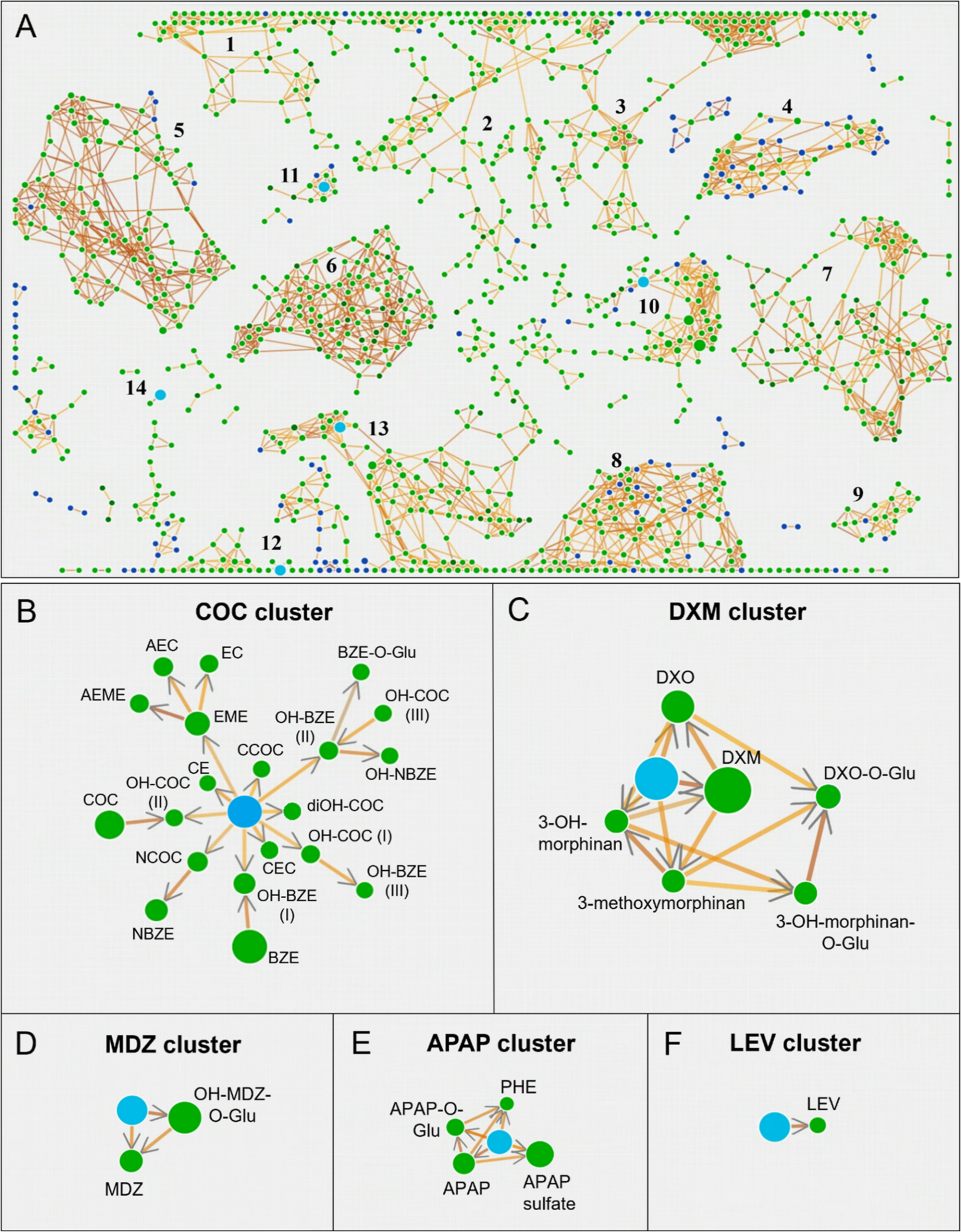
Transformation-aware molecular network of the urine data set. (A) Global network view. Compared to blood, the urine network shows a higher density and diversity of interconnected nodes, reflecting the broader range of metabolites excreted in this matrix. Global clusters correspond to endogenous and background biological chemistry, including medium-polarity aromatic compounds and glucoside-like derivatives (Cluster 1), amino acids and related small polar metabolites (Cluster 2), highly polar zwitterionic species such as betaine, trimethylamine-*N*-oxide, tranexamic acid, and stachydrine (Cluster 3), medium-hydrophobic amides and fatty acids (Cluster 4), multiple acylcarnitines (Clusters 5 and 8), oxidized fatty acid derivatives (Cluster 6), polyunsaturated fatty acids (Cluster 7), and sterol-related metabolites (Cluster 9). (B–F) Seed-based subnetworks for COC, DXM, MDZ, APAP, and LEV, illustrating each seed node together with the parent compounds and associated metabolites detected in urine. Abbreviations: 3-OH-morphinan, 3-hydroxymorphinan; 3-OH-morphinan-*O*-Glu, 3-hydroxymorphinan-*O*-glucuronide; AEC, anhydroecgonine; AEME, anhydroecgonine methyl ester; APAP, acetaminophen (paracetamol); APAP-*O*-Glu, acetaminophen-*O*-glucuronide; APAP sulfate, acetaminophen sulfate; BZE, benzoylecgonine; BZE-*O*-Glu, benzoylecgonine-*O*-glucuronide; CEC, cinnamoylecgonine; CCOC, cinnamoylcocaine; CE, cocaethylene; COC, cocaine; diOH–COC, dihydroxycocaine; DXM, dextromethorphan; DXO, dextrorphan; DXO-*O*-Glu, dextrorphan-*O*-glucuronide; EC, ecgonine; EME, ecgonine methyl ester; LEV, levamisole; MDZ, midazolam; NBZE, norbenzoylecgonine; NCOC, norcocaine; OH-BZE (I), hydroxybenzoylecgonine (isomer I); OH-BZE (II), hydroxybenzoylecgonine (isomer II); OH-BZE (III), hydroxybenzoylecgonine (isomer III); OH–COC (I), hydroxycocaine (isomer I); OH–COC (II), hydroxycocaine (isomer II); OH–COC (III), hydroxycocaine (isomer III); OH-MDZ-*O*-Glu, hydroxymidazolam-*O*-glucuronide; OH-NBZE, hydroxynorbenzoylecgonine; PHE, phenacetin.

Within this chemically complex feature space, seed-based subnetworks enabled focused interrogation of xenobiotic-related compounds and their metabolites (Figures 2B–F and 3B–F). In the blood data set, the COC seed subnetwork (Figure 2B) grouped the parent compound with BZE, NBZE, OH-BZE (two chromatographically resolved isomers), OH-NBZE, and OH–COC, and additionally connected ecgonine methyl ester (EME), ecgonine (EC), and anhydroecgonine (AEC). These latter compounds were not retained by the rule-based MetID filtering criteria, illustrating how network-based analysis can recover metabolite-related features that are not captured within the filtered rule-based annotation set. The DXM seed subnetwork in blood (Figure 2C) linked the parent compound exclusively to DXO, consistent with the limited metabolic signature observed in this matrix. In contrast, the MDZ seed subnetwork (Figure 2D) contained only the parent compound, reflecting the absence of detectable MDZ metabolites under the applied criteria. For LEV and APAP, no parent compound or metabolite-related features were detected in blood. Inclusion of the corresponding seed nodes in the network (Figure 2E,F) did not reveal any associated features, thereby confirming the absence of LEV- and APAP-related signals observed in the untargeted LC–HRMS and MetID analyses.

In urine, the molecular network exhibited a higher density of interconnected nodes, consistent with the increased diversity and abundance of metabolites excreted in this matrix. Seed-based subnetworks again enabled targeted interpretation of xenobiotic-related transformation pathways. The COC seed subnetwork (Figure 3B) incorporated all metabolites identified by MetID and additionally revealed further transformation products, including EME, EC, AEC,anhydroecgonine methyl ester (AEME), cinnamoylcocaine (CCOC), benzoylecgonine-*O*-glucuronide (BZE-*O*-Glu), and cocaethylene (CE). Formation of CE is consistent with ethanol-related transesterification chemistry and provides an analytically interpretable relationship between co-occurring features within the network. Chromatograms corresponding to all putative metabolites are shown in Figure 4.

**Figure 4:**
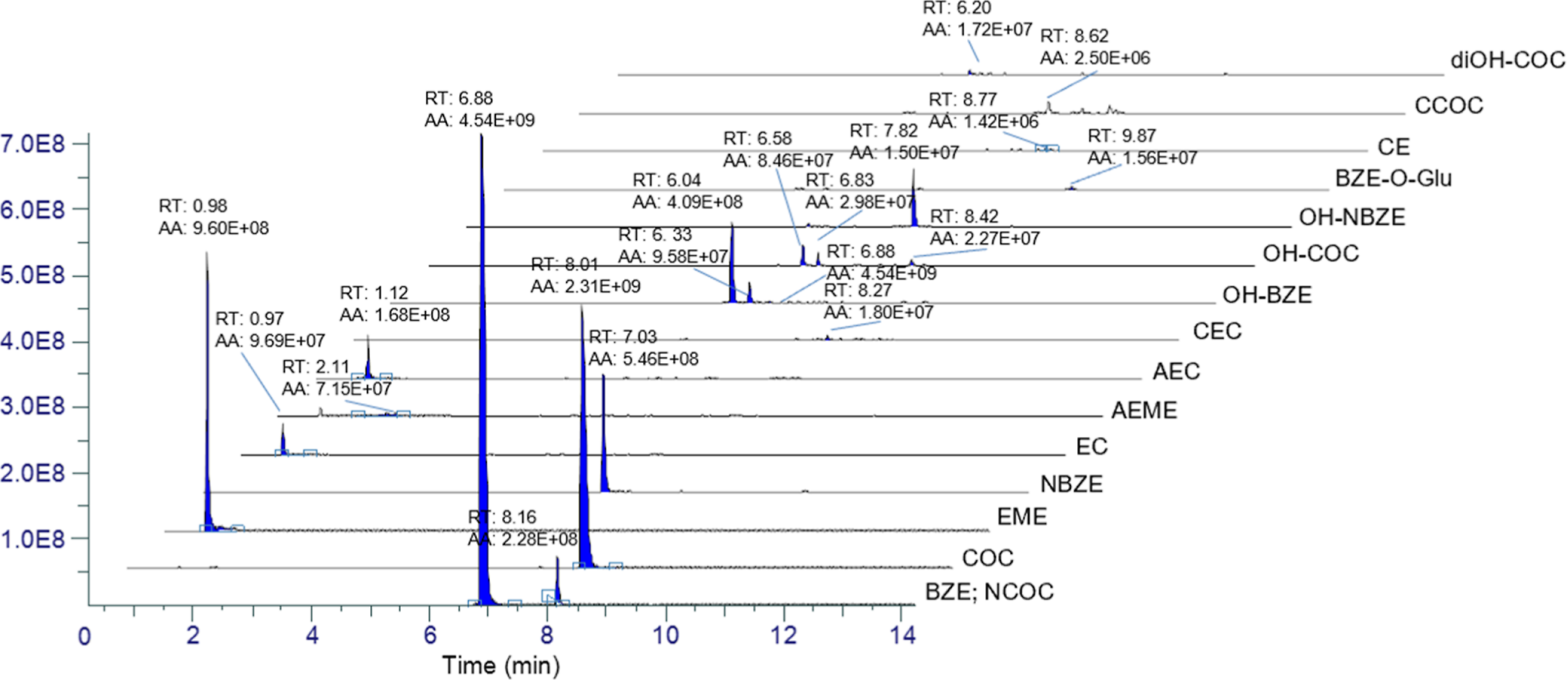
Overlay of extracted-ion chromatograms showing retention times of COC and its metabolites annotated in urine through transformation-aware MN, including oxidative, hydrolytic, conjugative, dehydrative, and ethanol-derived products. Abbreviations: AEC, anhydroecgonine; AEME, anhydroecgonine methyl ester; BZE, benzoylecgonine; BZE-*O*-Glu, benzoylecgonine-*O*-glucuronide; CEC, cinnamoylecgonine; CCOC, cinnamoylcocaine; CE, cocaethylene; COC, cocaine; diOH–COC, dihydroxycocaine; EC, ecgonine; EME, ecgonine methyl ester; NBZE, norbenzoylecgonine; NCOC, norcocaine; OH-BZE, hydroxybenzoylecgonine; OH–COC, hydroxycocaine; OH-NBZE, hydroxynorbenzoylecgonine.

The DXM seed subnetwork in urine (Figure 3C) connected the parent compound to phase I metabolites and phase II conjugates, in agreement with MetID-based annotations. The MDZ seed subnetwork (Figure 3D) grouped the parent compound with OH-MDZ-*O*-Glu, while the APAP seed subnetwork (Figure 3E) linked APAP-*O*-Glu and APAP sulfate and showed connectivity with phenacetin (PHE). This network relationship is compatible with an adulterant-derived origin of APAP-related signals rather than direct administration. The LEV seed subnetwork (Figure 3F) contained the parent compound without detectable metabolites.

A quantitative comparison of rule-based and network-based annotation outcomes is summarized in Figure 5. MetID annotated 10 compounds in blood and 25 in urine, all of which were recovered by molecular networking. Transformation-aware molecular networking further connected additional metabolite-related features, increasing the total number of annotated compounds to 13 in blood and 33 in urine. These additional features were linked through consistent spectral similarity and biotransformation relationships, supporting their contextual interpretation within the molecular network.

**Figure 5:**
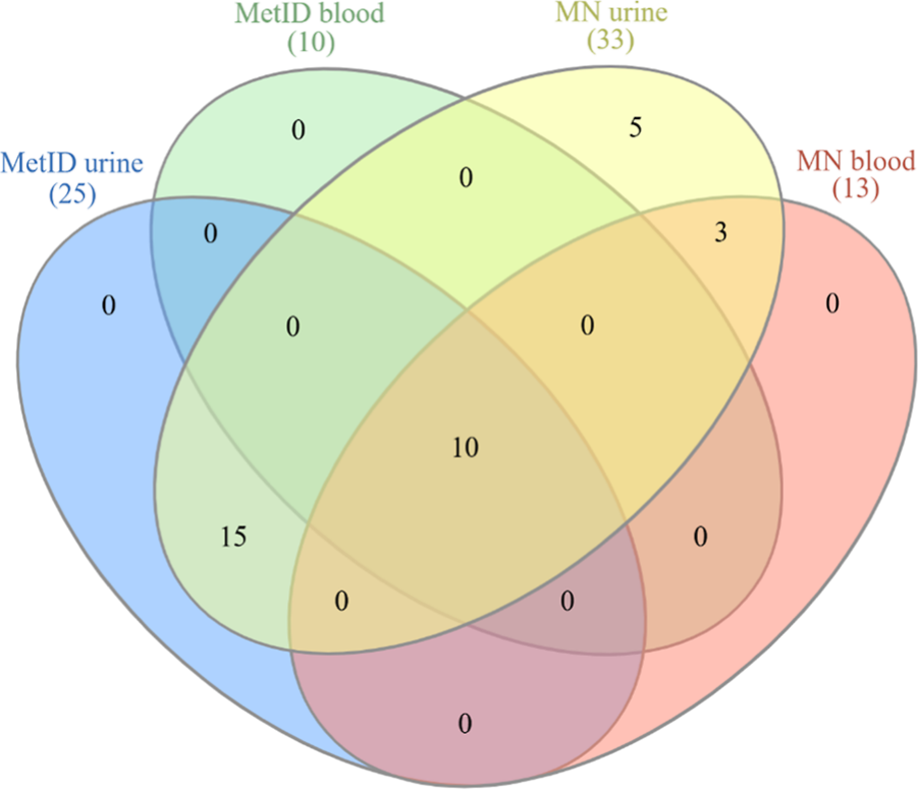
Venn diagram illustrating the overlap between compounds annotated by MetID in blood and urine and those recovered through transformation-aware molecular networking (MN) in the same matrices. Counts include parent compounds and their putative metabolites. MetID identified 10 compounds in blood and 25 in urine, all of which were recovered by MN. MN annotated 13 compounds in blood and 33 in urine, revealing additional metabolites not captured by MetID and highlighting the broader coverage of the network-based approach.

Overall, transformation-aware molecular networking complemented rule-based metabolite prediction by extending metabolite annotation beyond the filtered rule-based set and by providing a relational framework for interpreting xenobiotics, metabolites, and adulterant-related species within untargeted LC–HRMS data sets. A comprehensive overview of all seed-related features is provided in Tables S8–S11, including compound-level parameters (*m*/*z*, retention time, exact mass, Δ*m*/*z*, peak area, annotation metrics, and identification confidence levels assigned according to the Schymanski framework; Section 2.9) as well as network-specific similarity metrics supporting node connectivity. The corresponding experimental MS/MS fragmentation spectra, including annotated fragment ions, are provided in the Supporting Information (Figures S3–S48).

## 4 Discussion

### 4.1 Conceptual Distinction from Conventional Molecular Networking

While classical and feature-based molecular networking (CMN and FBMN) approaches^2,13^ have significantly improved relational interpretation of untargeted LC–HRMS data, network connectivity in these strategies remains primarily driven by MS/MS spectral similarity. As noted in the Introduction, similarity alone may not reliably reconstruct coherent biotransformation pathways, particularly when structurally divergent or low-abundance metabolites are involved.

The framework presented here addresses this limitation by embedding curated phase I and phase II biotransformation rules directly into network construction and shortest-path evaluation. Connectivity is therefore conditioned not only on spectral similarity but also on mechanistic plausibility and relational consistency. This transformation-constrained logic shifts molecular networking from descriptive clustering toward structured reconstruction of metabolically coherent families anchored to known xenobiotics.

In the forensic context examined in this study, such integration proved particularly relevant for contextualizing metabolites of co-occurring substances within complex biological matrices. By enforcing biochemical consistency in addition to spectral similarity, the workflow enhances interpretability in scenarios where parent compounds may be partially absent, present at low abundance, or masked by adulterants.

### 4.2 Analytical Robustness and Edge Specificity

Robust interpretation of untargeted LC–HRMS data requires careful control of spurious feature associations. In the present framework, edge formation relied on multilayer constraints including MS/MS similarity thresholds, biologically restricted transformation rules, and shortest-path consistency. Mass differences alone were not considered sufficient to establish connectivity. Limiting transformation rules to physiologically plausible xenobiotic phase I and phase II reactions reduced the likelihood that unrelated endogenous features would satisfy transformation criteria.

Potential analytical artifacts were explicitly evaluated. No reported metabolite coeluted with its corresponding parent compound, reducing the probability of in-source fragmentation artifacts. Conventional HESI parameters were applied, and no evidence of electrospray-induced reaction products^18^ was observed under the analytical conditions employed. Together, these constraints support the specificity and robustness of the resulting network architecture.

### 4.3 Limitations and Generalizability

This study represents a proof-of-concept application based on a single DUID case and two biological matrices acquired under positive electrospray ionization conditions. Although the framework demonstrated interpretative advantages under realistic forensic conditions, broader validation across additional cases and compound panels is warranted to further assess robustness and performance boundaries.

The current implementation was conducted within the Compound Discoverer 3.4 environment using high-resolution accurate-mass data acquired on a Thermo Orbitrap platform. Consequently, the operational workflow is specific to this software ecosystem. However, the underlying transformation-aware logic—based on MS/MS spectral similarity, curated biotransformation constraints, and relational path consistency—is conceptually transferable to other HRMS data-processing environments that enable comparable integration of these elements.

Parameter selection, including mass tolerance and spectral similarity thresholds, primarily influences network density rather than the underlying transformation logic. Conservative thresholds enhance specificity, whereas relaxed settings increase connectivity at the potential cost of spurious associations. Future work will evaluate the framework across expanded data sets, additional ionization modes, and alternative analytical platforms, as well as through continued refinement of spectral libraries and biotransformation rule sets. Integration of additional quantitative metrics and isotope-pattern information may further enhance confidence assessment in transformation-aware network interpretation.

## 5 Conclusions

In this study, untargeted LC–HRMS data were interpreted by integrating rule-based metabolite prediction with transformation-aware molecular networking, enabling relational organization of parent compounds, metabolites, and structurally related features within a single analytical framework. This combined approach improved metabolite coverage and supported contextual interpretation of xenobiotic-related signals in complex biological matrices.

Applied to blood and urine samples from a representative forensic case, the framework confirmed multiple drugs of abuse and therapeutics and enabled reconstruction of a chemically consistent cocaine metabolic profile, including low-abundance metabolites not retained by rule-based annotation alone. Network-based analysis further supported interpretation of ethanol-related cocaethylene formation and revealed a metabolically consistent association between acetaminophen and phenacetin, demonstrating how relational analysis can provide additional contextual insight beyond conventional untargeted workflows.

Overall, transformation-aware molecular networking is presented here as a complementary analytical layer that links MS/MS spectral similarity with biotransformation logic to support structured interpretation of untargeted LC–HRMS data sets. This framework provides a generalizable strategy for organizing and interpreting complex mass spectrometry data in forensic and toxicological investigations, with relevance for routine analysis of chemically complex samples.

## Supplementary Material



## Notes

Untargeted LC–HRMS data-processing workflow implemented in Compound Discoverer (CD) (Figure S1), extended data-processing workflow incorporating MetID and transformation-aware molecular networking (Figures S2), list of Phase I and Phase II biotransformations used for metabolite prediction in MetID and for transformation-aware Molecular Networking (Table S1), diagnostic fragment ions used for Class Compound Scoring of the COC, DXM, MDZ, LEV, and APAP seed (Tables S2–S6), experimental MS/MS fragmentation spectra of reported blood and urine metabolites, including annotated fragment ions and putative structural assignments where applicable (Figures S3–S48), overview of all MetID-proposed metabolites, including their predicted structures and associated phase I/II metabolic transformations (Table S7), lists of parent compounds and associated blood metabolites identified within the transformation-aware molecular networking workflow (Table S8), together with the corresponding network-derived connectivity metrics for blood metabolites (Table S9), lists of parent compounds and associated urine metabolites identified within the transformation-aware molecular networking workflow (Table S10), together with the corresponding network-derived connectivity metrics for urine metabolites (Table S11) (PDF)
