# Supplementary material for: Transformation-Aware Molecular Networking for Interpretation of Untargeted LC–HRMS Data
Source: ACS Meas Sci Au. 2026 Mar 26;6(3):703–14. doi: 10.1021/acsmeasuresciau.6c00011 (PMC13281190; doi:10.1021/acsmeasuresciau.6c00011)
Supplement: Supplementary file 1 [file tg6c00011_si_001.pdf]

Supporting Information

Transformation-Aware Molecular Networking for Interpretation of Untargeted LC–HRMS Data

Elena Ferri,[a,b,*] Cristian Caprari,[a,c] Maria Angela Vandelli,[a] Rossana Cecchi,[d] Patrizia Verri,[d] Giuseppe Cannazza,[a,e] Cinzia Citti,[e,a,*]

[a] Department of Life Sciences, University of Modena and Reggio Emilia, Via Campi 103, 41125 – Modena, Italy

[b] Health Innovative Products and Technologies (HIP-TECH) PhD Program, Department of Life Sciences, University of Modena and Reggio Emilia, 41125 – Modena, Italy

[c] Clinical and Experimental Medicine (CEM) PhD Program, Nanomedicine, Medicinal and Pharmaceutical Sciences, Department of Life Sciences, University of Modena and Reggio Emilia, 41125 – Modena, Italy

[d] Department of Biomedical, Metabolic and Neural Sciences, Institute of Legal Medicine, University of Modena and Reggio Emilia, 41125 – Modena, Italy

[e] Institute of Nanotechnology – CNR NANOTEC, Campus Ecotekne, Via Monteroni, 73100 – Lecce, Italy

***Corresponding authors:**
Dr. Elena Ferri, phone: +39 059 2058575; e-mail: elena.ferri@unimore.it
Dr. Cinzia Citti, Ph.D., phone: +39 0832 319206; e-mail: cinzia.citti@cnr.it

Table of contents

Figure S1: Untargeted LC–HRMS data-processing workflow implemented in Compound Discoverer (CD)	3
Figure S2: Extended data-processing workflow incorporating MetID and transformation-aware molecular networking	4
Table S1: Phase I and Phase II biotransformations used for metabolite	5

prediction in MetID and for transformation-aware Molecular Networking	
Table S2: Diagnostic fragment ions used for Class Compound Scoring of the COC seed	6
Table S3: Diagnostic fragment ions used for Class Compound Scoring of the DXM seed	7
Table S4: Diagnostic fragment ions used for Class Compound Scoring of the MDZ seed	9
Table S5: Diagnostic fragment ions used for Class Compound Scoring of the LEV seed	10
Table S6: Diagnostic fragment ions used for Class Compound Scoring of the APAP seed	12
Figures S3-S15: Experimental MS/MS fragmentation spectra of reported blood metabolites, including annotated fragment ions and putative structural assignments where applicable	14
Figures S16-S48: Experimental MS/MS fragmentation spectra of reported urine metabolites, including annotated fragment ions and putative structural assignments where applicable	20
Table S7: Overview of all MetID-proposed metabolites, including their predicted structures	37

and associated phase I/II metabolic transformations	
Table S8: List of parent compounds and associated blood metabolites identified within the transformation-aware molecular networking workflow	38
Table S9: List of network-derived connections for blood metabolites identified within the transformation-aware molecular network	39
Table S10: List of parent compounds and associated urine metabolites identified within the transformation-aware molecular networking workflow	39
Table S11: List of network-derived connections for urine metabolites identified within the transformation-aware molecular network	41

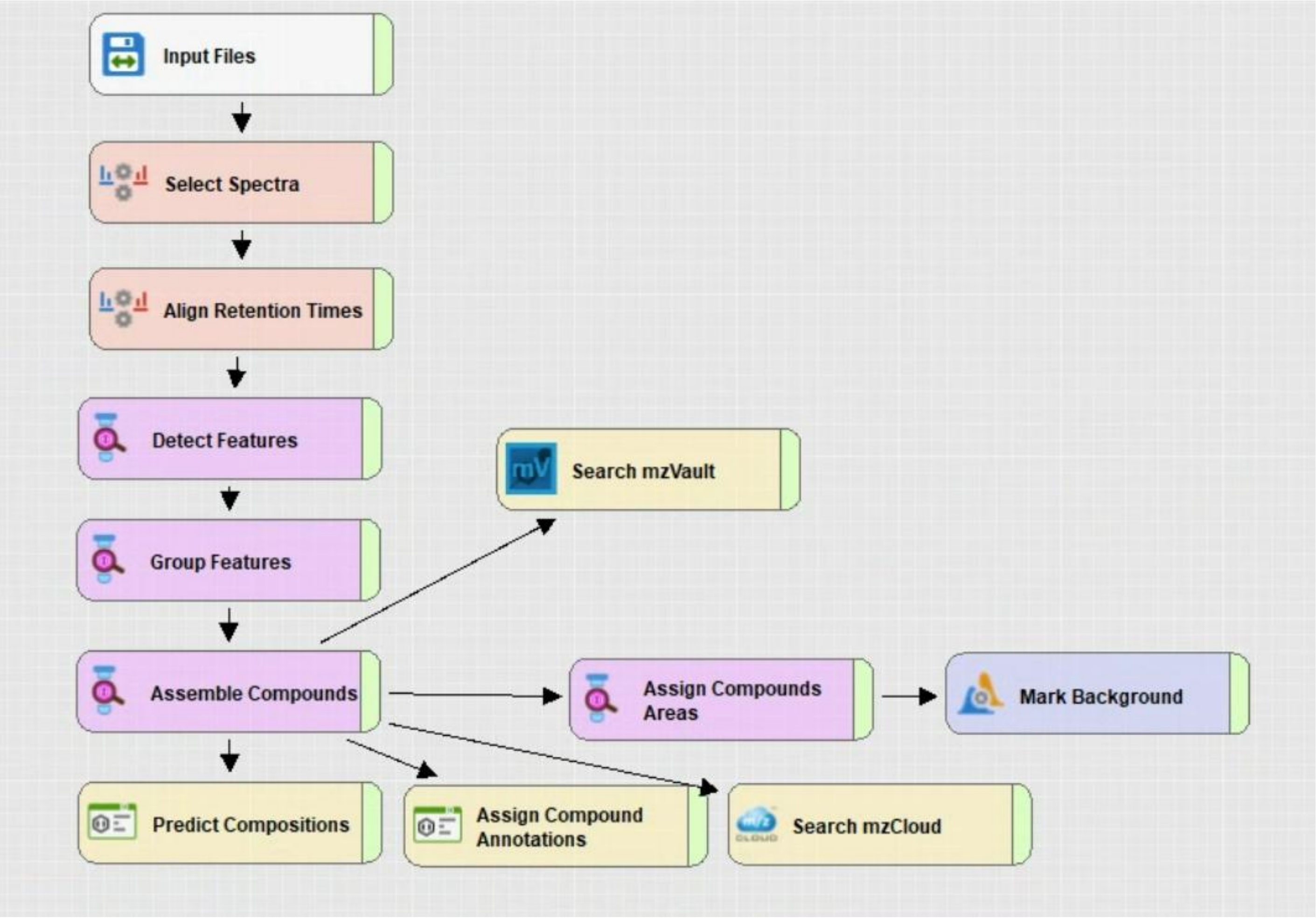

Figure S 1 Untargeted LC–HRMS data-processing workflow implemented in Compound Discoverer (CD). *The workflow includes retention time alignment, feature detection, feature grouping, adduct assignment, compound assembly, spectral library searching (mzCloud and mzVault), elemental composition prediction, and background subtraction. All steps used for untargeted feature annotation in blood and urine are shown.*

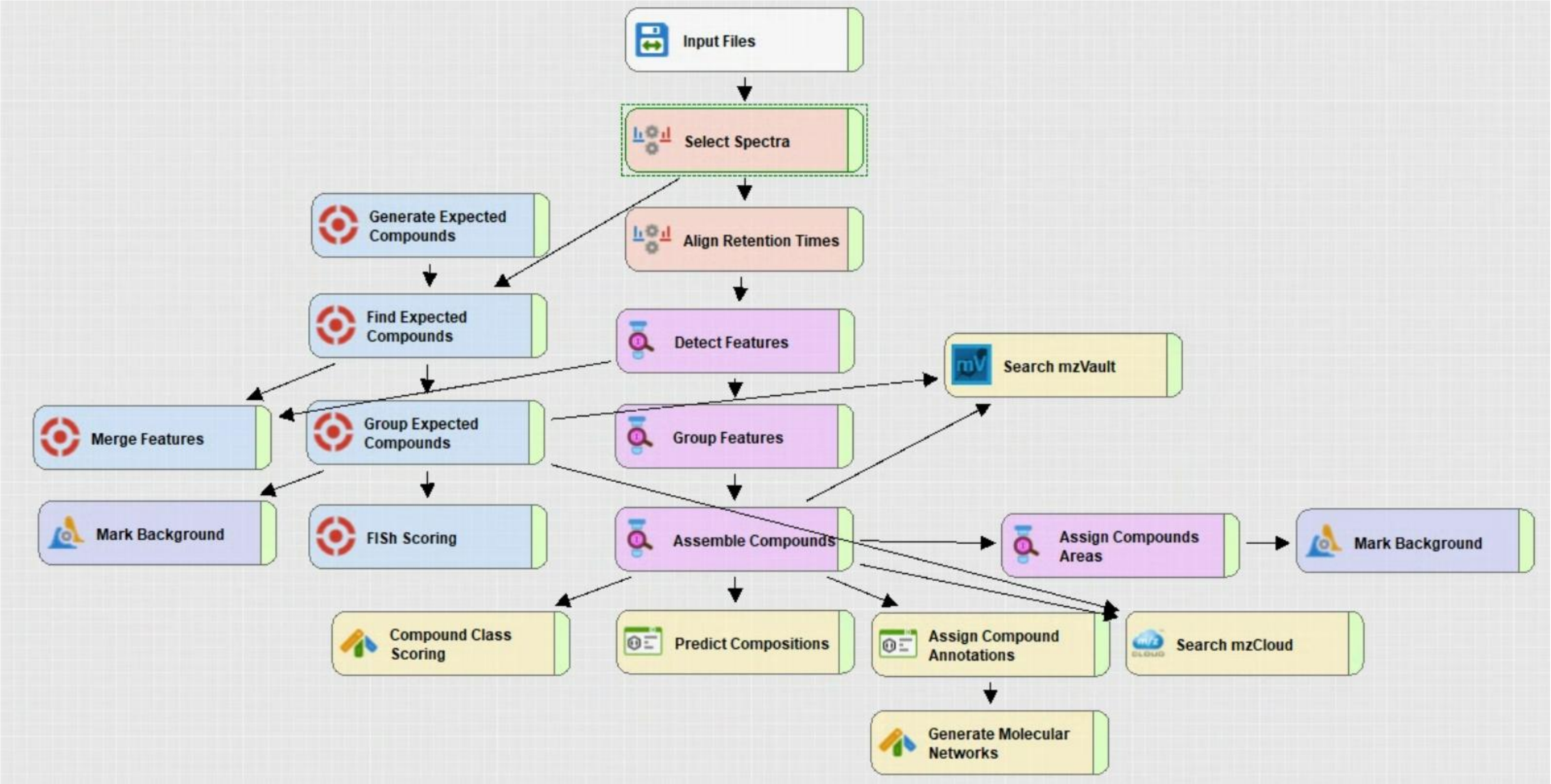

Figure S 2 Extended data-processing workflow incorporating MetID and transformation-aware molecular networking. *This workflow builds upon the untargeted pipeline shown in Figure S1 through the addition of MetID-specific nodes ("Generate Expected Compounds," "Find Expected Compounds," "Group Expected Compounds," "FISh Scoring," and "Merge Features") and the "Generate Molecular Networking" node. These additions enable the prediction, detection, and structural correlation of metabolites through both MS² spectral similarity and biotransformation-based relationships.*

Table S 1 Phase I (A) and Phase II (B) biotransformations used for metabolite prediction in MetID and for transformation-aware Molecular Networking

A

**Transformation Type**	**Transformation**	**Formula Change**
Phase I	Dehydration	$-H_2O$
Phase I	Demethylation	$-CH_3$
Phase I	Desaturation	$-H_2$
Phase I	Hydration	$+H_2O$
Phase I	Nitro Reduction	$-NO_2$ $+NH_2$
Phase I	Oxidation	+O
Phase I	Oxidative Deamination to Alcohol	$-NH_2$ +OH
Phase I	Oxidative Deamination to Ketone	$-NH_3$ +O
Phase I	Oxidative Debromination	–Br +OH
Phase I	Oxidative Dechlorination	–Cl +OH
Phase I	Oxidative Defluorination	–F +OH
Phase I	Reduction	$+H_2$
Phase I	Reductive Debromination	–Br +H
Phase I	Reductive Dechlorination	–Cl +H
Phase I	Reductive Defluorination	–F +H
Phase I	Thiourea → Urea Conversion	–S +O

B

**Transformation Type**	**Transformation**	**Formula Change**
Phase II	Acetylation	$+C_2H_3O$
Phase II	Arginine Conjugation	$+C_6H_{13}N_4O_2$
Phase II	Cysteine Conjugation I	$+C_3H_6NO_2S$
Phase II	Cysteine Conjugation II	$+C_3H_7NO_2S$
Phase II	Glucoside Conjugation	$+C_6H_{11}O_5$
Phase II	Glucuronide Conjugation	$+C_6H_9O_6$
Phase II	Glutamine Conjugation	$+C_5H_9N_2O_3$
Phase II	Glycine Conjugation	$+C_2H_4NO_2$
Phase II	GSH Conjugation (Br)	$+C_{10}H_{16}N_3O_6S$
Phase II	GSH Conjugation (Cl)	$+C_{10}H_{16}N_3O_6S$
Phase II	GSH Conjugation (F)	$+C_{10}H_{16}N_3O_6S$
Phase II	GSH Conjugation I	$+C_{10}H_{15}N_3O_6S$
Phase II	GSH Conjugation II	$+C_{10}H_{17}N_3O_6S$
Phase II	Methylation	$+CH_3$
Phase II	Ornithine Conjugation	$+C_5H_{11}N_2O_2$
Phase II	Palmitoyl Conjugation	$+C_{16}H_{31}O$
Phase II	Stearyl Conjugation	$+C_{18}H_{35}O$
Phase II	Sulfation	$+SO_3$
Phase II	Taurine Conjugation	$+C_2H_6NO_3S$

Table S 2 Diagnostic fragment ions used for Class Compound Scoring of the cocaine (COC) seed. *The table lists the characteristic MS/MS fragments selected to recognize COC and its structurally related metabolites within the molecular network.*

m/z $[M+H]^+$	Structure $[M+H]^+$	Formula $[M+H]^+$
82.0651	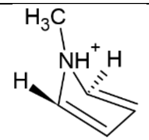	C_5H_8N
150.0913	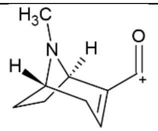	$C_9H_{13}NO$
168.1019	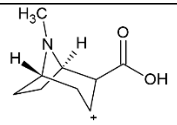	$C_9H_{14}NO_2$
182.1175	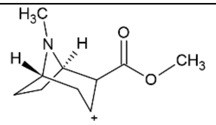	$C_{10}H_{16}NO_2$
186.1126	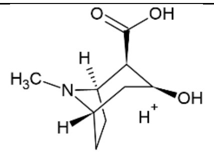	$C_9H_{16}NO_3$
290.1387	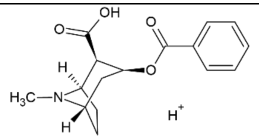	$C_{16}H_{20}NO_4$
304.1540	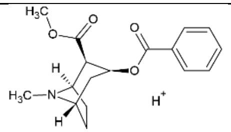	$C_{17}H_{22}NO_4$

Table S 3 Diagnostic fragment ions used for Class Compound Scoring of the dextromethorphan (DXM) seed. *Reported fragments were used to identify DXM and its phase I/II metabolites within the transformation-aware MN workflow.*

m/z $[M+H]^+$	Structure $[M+H]^+$	Formula $[M+H]^+$
121.0649	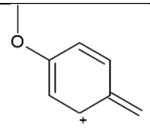	C_8H_9O
145.0650	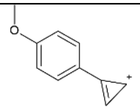	$C_{10}H_9O$
147.0804	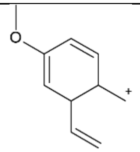	$C_{10}H_{13}O$
159.0805	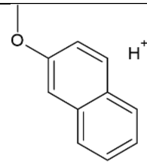	$C_{11}H_{11}O$
171.0800	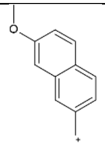	$C_{12}H_{11}O$
173.0961	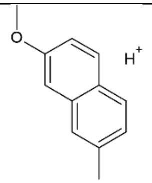	$C_{12}H_{13}O$
213.1273	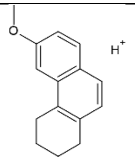	$C_{15}H_{17}O$

215.143	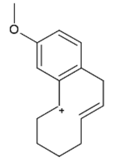	$C_{15}H_{19}O$
241.1585	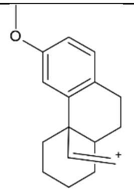	$C_{17}H_{21}O$
272.2007	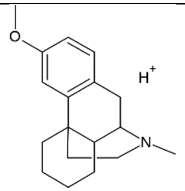	$C_{18}H_{26}NO$

Table S 4 Diagnostic fragment ions selected for the midazolam (MDZ) seed. *These fragments support recognition of MDZ-related features in the molecular network, including hydroxylated and conjugated derivatives.*

**m/z [M+H]$^+$**	**Structure [M+H]$^+$**	**Formula [M+H]$^+$**
244.0328	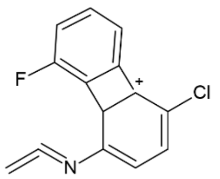	$C_{14}H_8ClFN$
285.0584	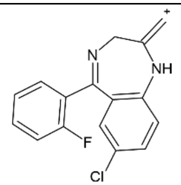	$C_{16}H_{11}ClFN_2$
290.1094	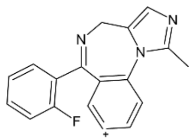	$C_{18}H_{13}FN_3$
291.1165	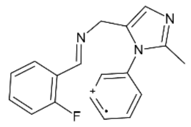	$C_{18}H_{14}FN_3$
326.0855	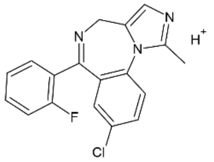	$C_{18}H_{14}ClFN_3$

Table S 5 Diagnostic fragment ions used for Class Compound Scoring of the levamisole (LEV) seed. *These fragments were applied to detect LEV-related nodes and discriminate them from unrelated features within the molecular network.*

m/z $[M+H]^+$	Structure $[M+H]^+$	Formula $[M+H]^+$
88.02207	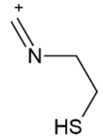	C_3H_6NS
91.05466	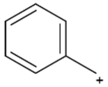	C_7H_7
105.0703	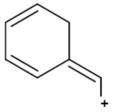	C_8H_9
117.0574	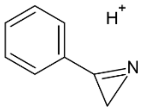	C_8H_7N
117.0702	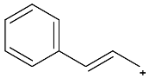	C_9H_9
118.0652	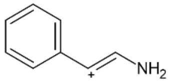	C_8H_8N
129.0699	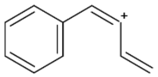	$C_{10}H_9$
145.0758	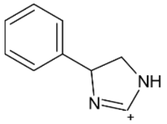	$C_9H_9N_2$

146.0965	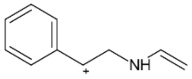	$C_{10}H_{12}N$
150.0370	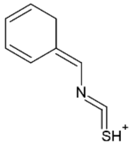	C_8H_8NS
178.0685	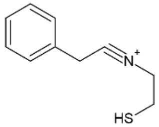	$C_{11}H_{10}NS$
188.0531	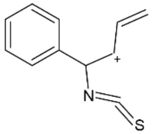	$C_{10}H_{12}NS$
205.0794	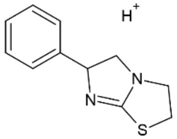	$C_{11}H_{13}N_2S$

Table S 6 Diagnostic fragment ions used for Class Compound Scoring of the acetaminophen (APAP) seed. *Listed ions facilitate annotation of APAP and its phase II metabolites in the MN-derived clusters.*

**m/z [M+H]$^+$**	**Structure [M+H]$^+$**	**Formula [M+H]$^+$**
65.03936	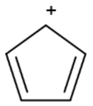	C_5H_5
92.0500	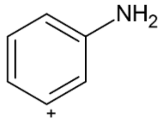	C_6H_6N
93.0579	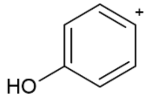	C_6H_5O
110.0604	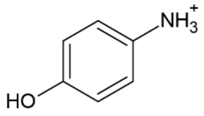	C_6H_8NO
111.0444	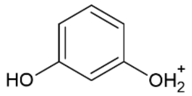	$C_6H_7O_2$
134.0602	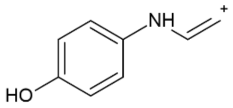	$C_8H_{10}NO$
152.0707	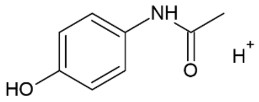	C_8H_8NO

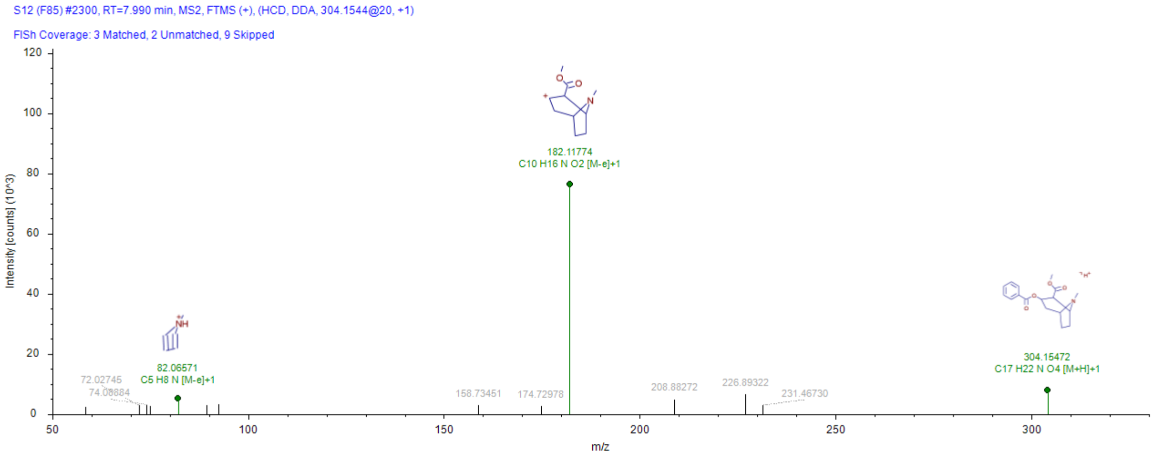

Figure S 3 *Experimental MS/MS fragmentation spectrum of cocaine ($[M+H]^+$, m/z 304.1544) detected in blood sample.*

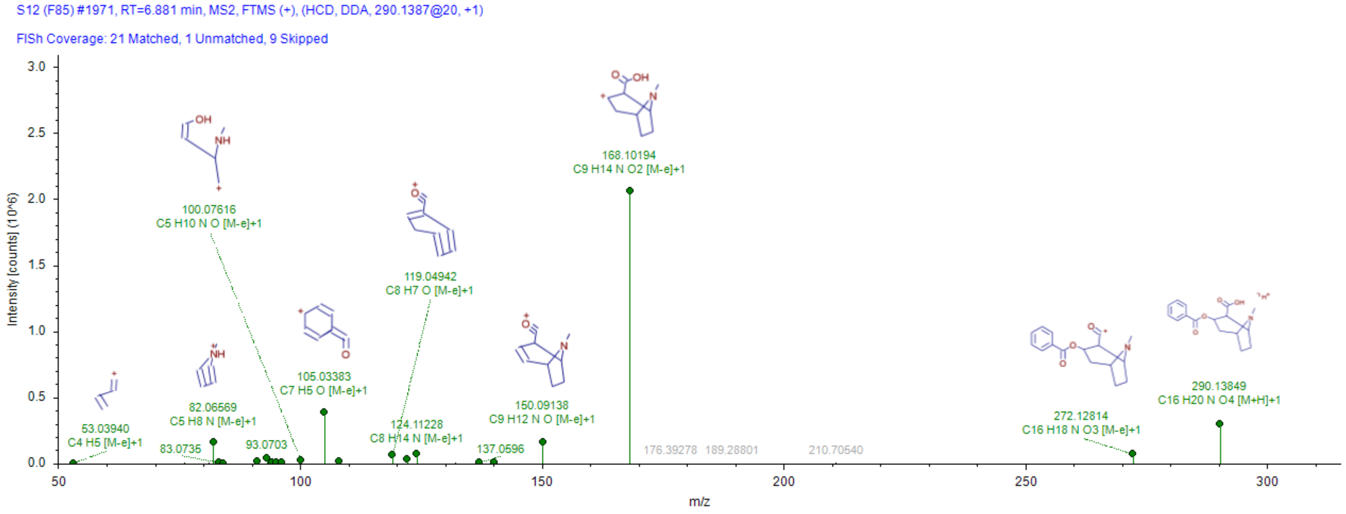

Figure S 4 *Experimental MS/MS fragmentation spectrum of benzoylecgonine ($[M+H]^+$, m/z 290.1387) detected in blood sample.*

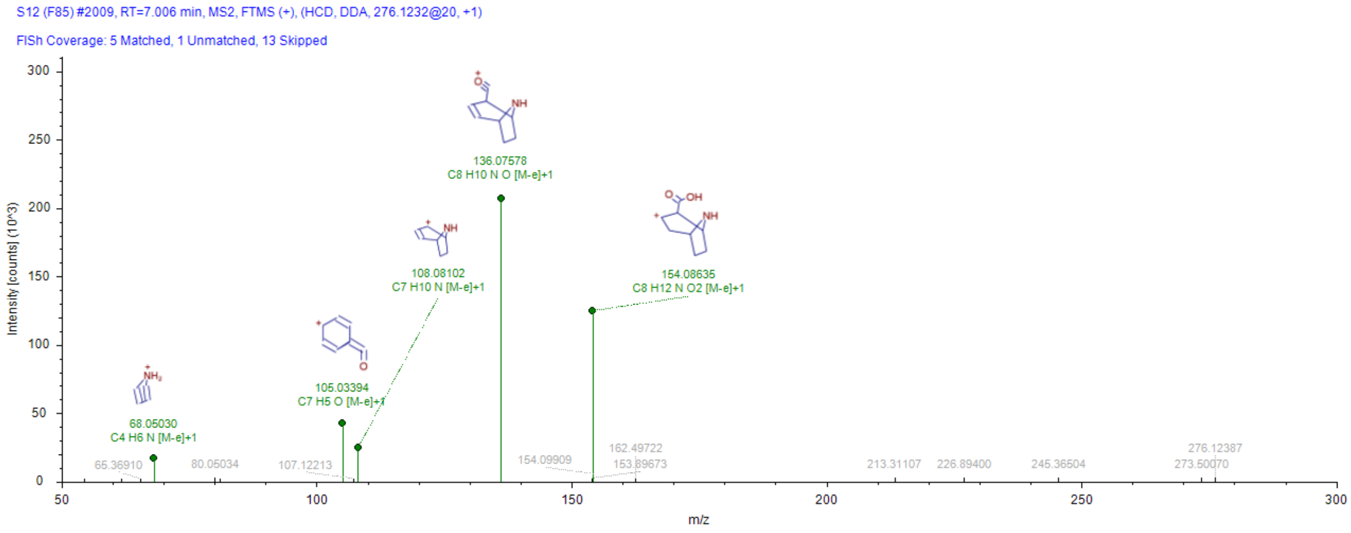

Figure S 5 *Experimental MS/MS fragmentation spectrum of norbenzoylecgonine ([M+H]⁺, m/z 276.1232) detected in blood sample.*

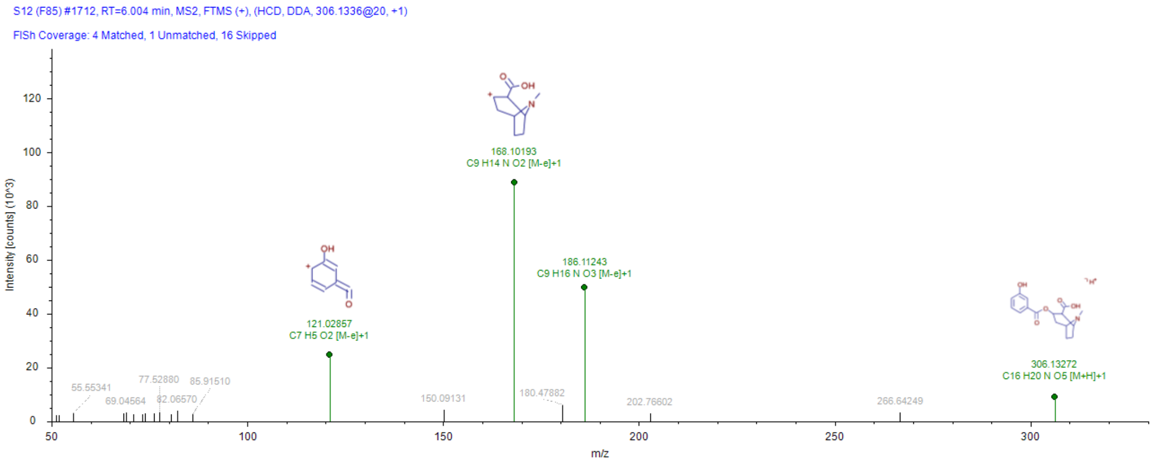

Figure S 6 *Experimental MS/MS fragmentation spectrum of hydroxybenzoylecgonine (isomer I) ([M+H]⁺, m/z 306.1336) detected in blood sample.*

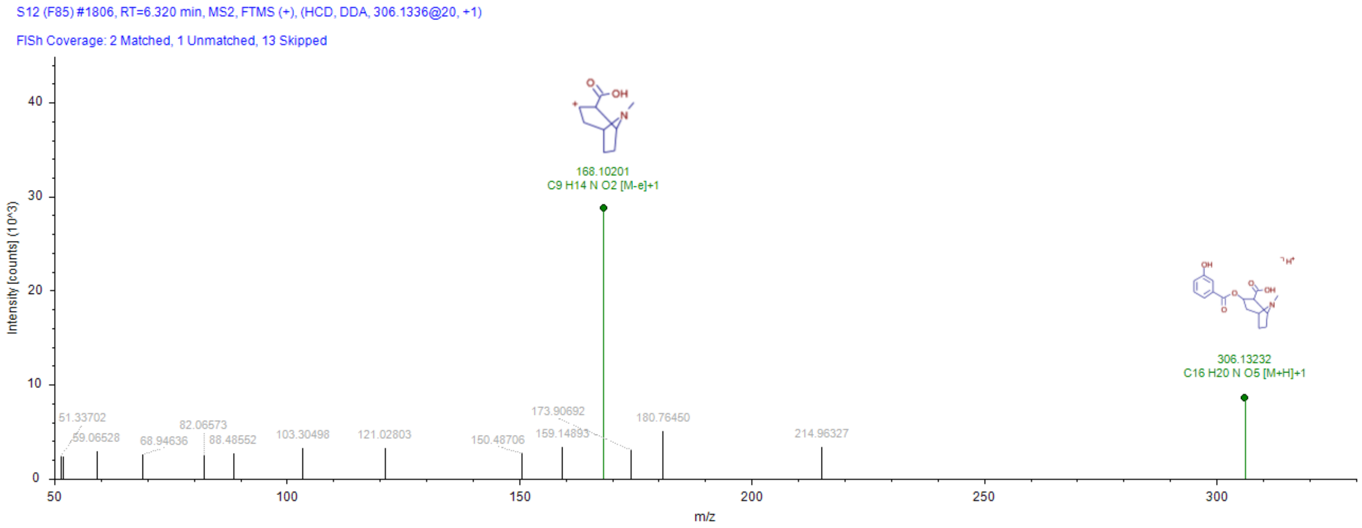

Figure S 7 *Experimental MS/MS fragmentation spectrum of hydroxybenzoylecgonine (isomer II) ($[M+H]^+$, m/z 306.1336) detected in blood sample.*

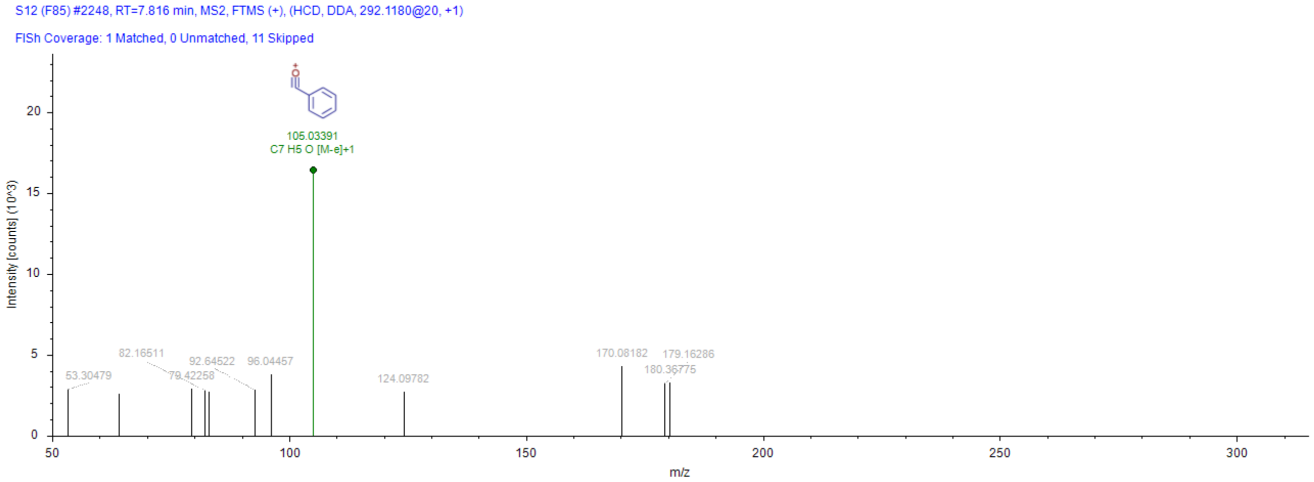

Figure S 8 *Experimental MS/MS fragmentation spectrum of hydroxynorbenzoylecgonine ($[M+H]^+$, m/z 292.1180) detected in blood sample.*

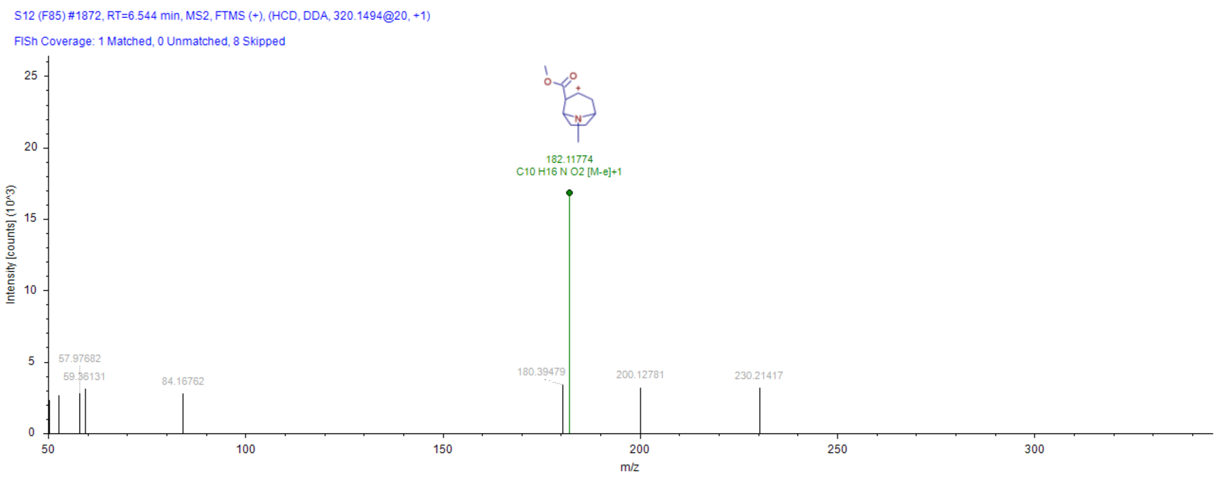

Figure S 9 *Experimental MS/MS fragmentation spectrum of hydroxycocaine ([M+H]$^+$, m/z 320.1494) detected in blood sample.*

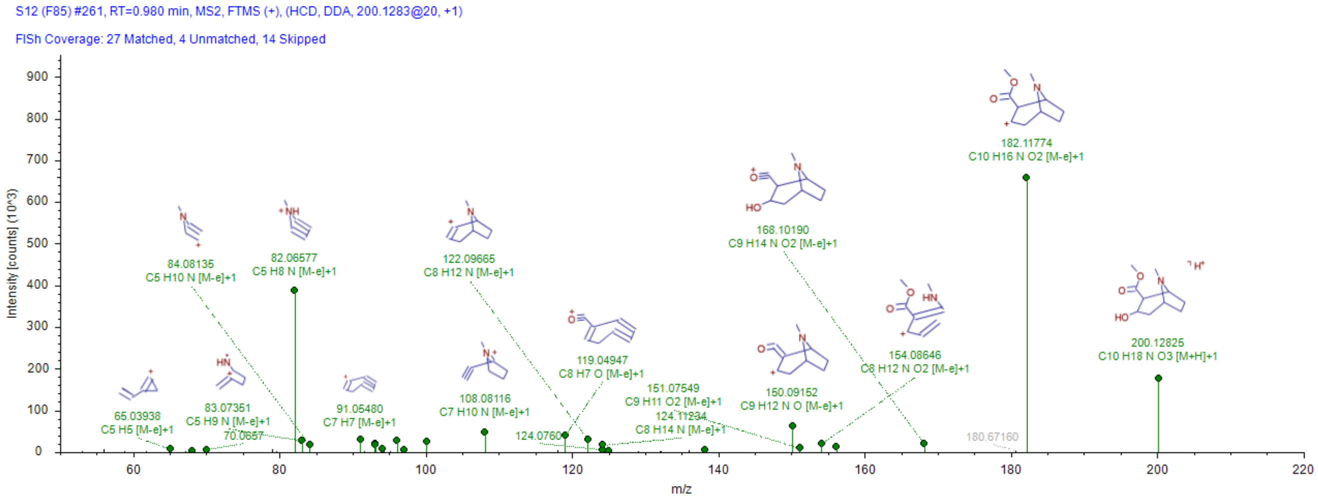

Figure S 10 *Experimental MS/MS fragmentation spectrum of ecgonine methyl ester ([M+H]$^+$, m/z 200.1283) detected in blood sample.*

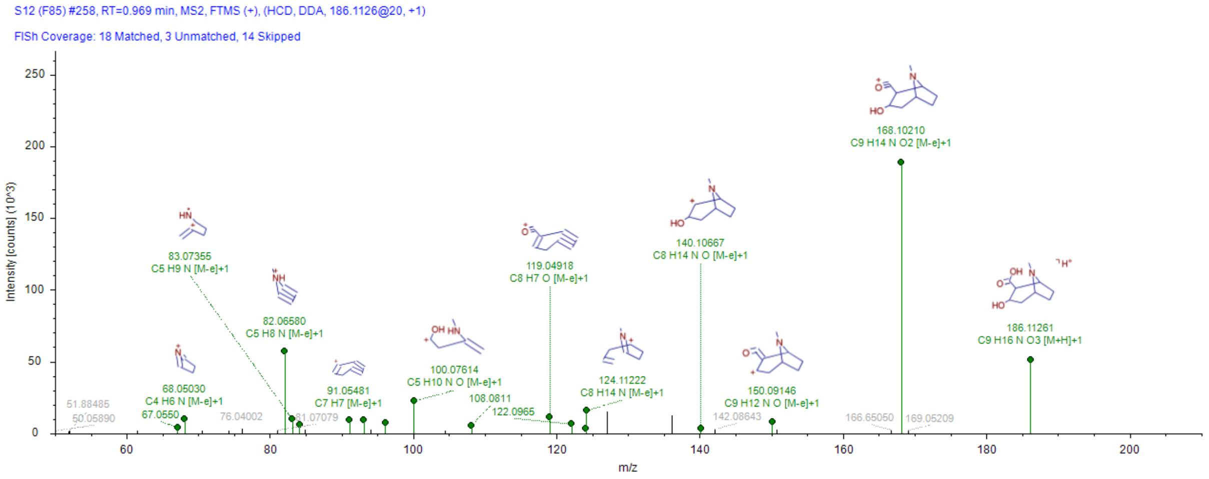

Figure S 11 *Experimental MS/MS fragmentation spectrum of ecgonine ([M+H]⁺, m/z 186.1126) detected in blood sample.*

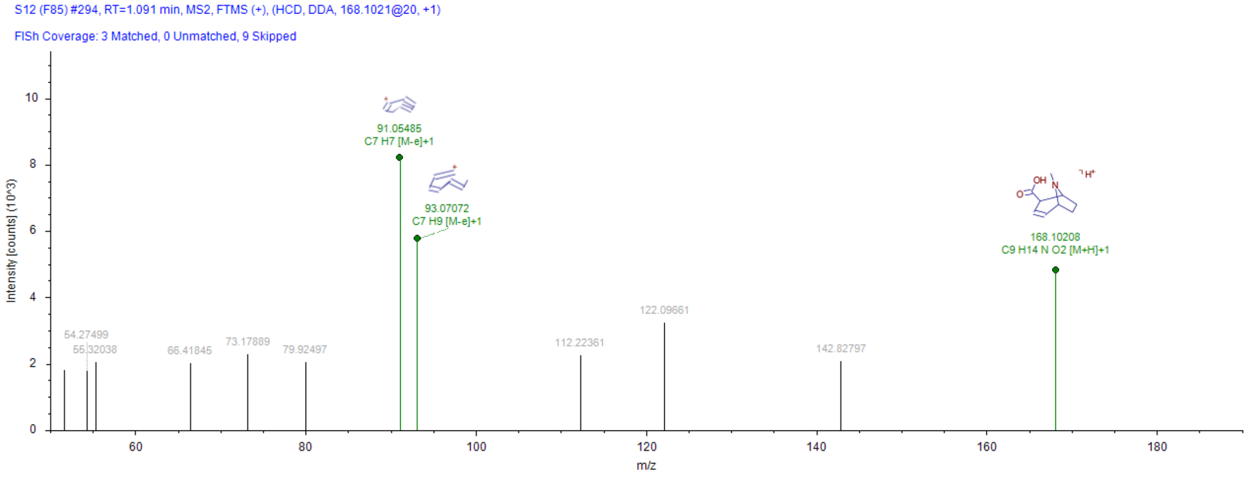

Figure S 12 *Experimental MS/MS fragmentation spectrum of anhydroecgonine ([M+H]⁺, m/z 168.1021) detected in blood sample.*

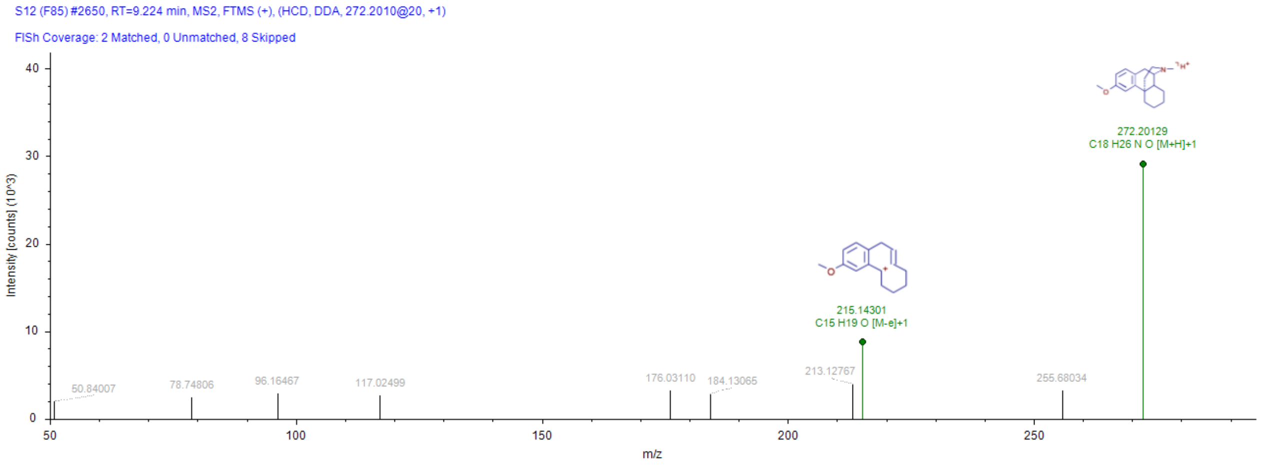

Figure S 13 *Experimental MS/MS fragmentation spectrum of dextromethorphan ([M+H]⁺, m/z 272.2010) detected in blood sample.*

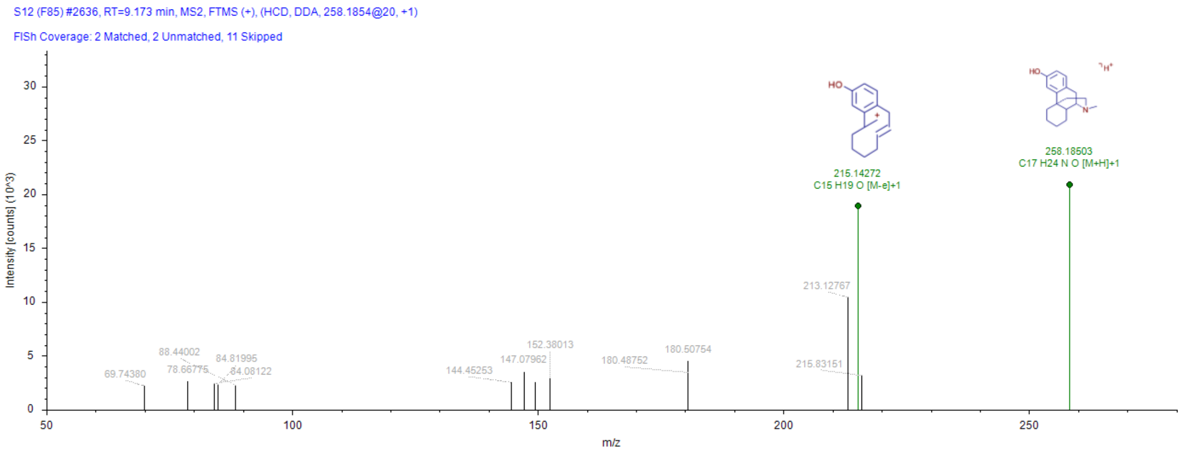

Figure S 14 *Experimental MS/MS fragmentation spectrum of dextrorphan ([M+H]⁺, m/z 258.1854) detected in blood sample.*

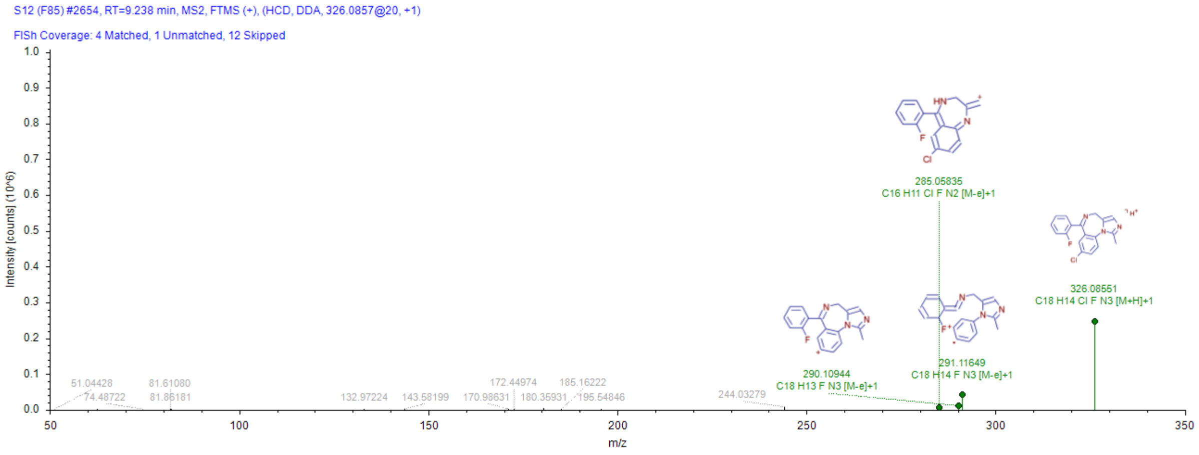

Figure S 15 *Experimental MS/MS fragmentation spectrum of midazolam ([M+H]$^+$, m/z 326.0857) detected in blood sample.*

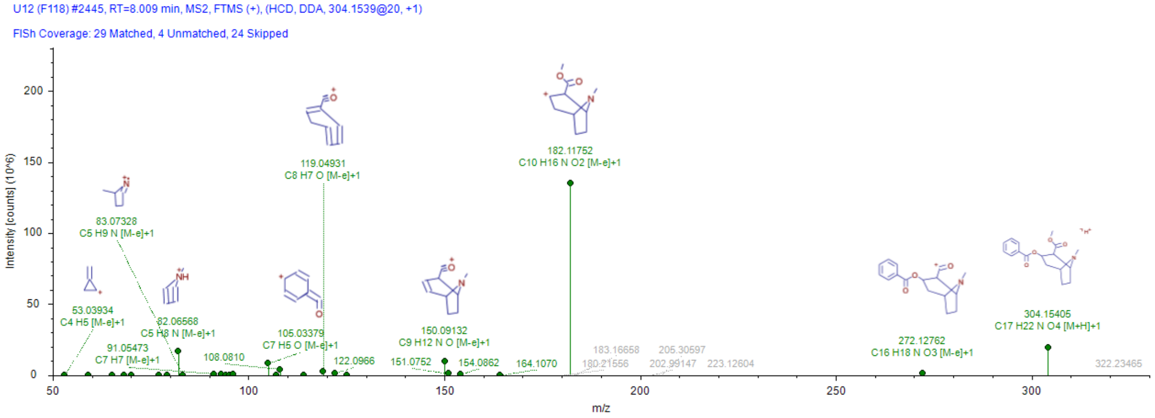

Figure S 16 *Experimental MS/MS fragmentation spectrum of cocaine ([M+H]$^+$, m/z 304.1539) detected in urine sample.*

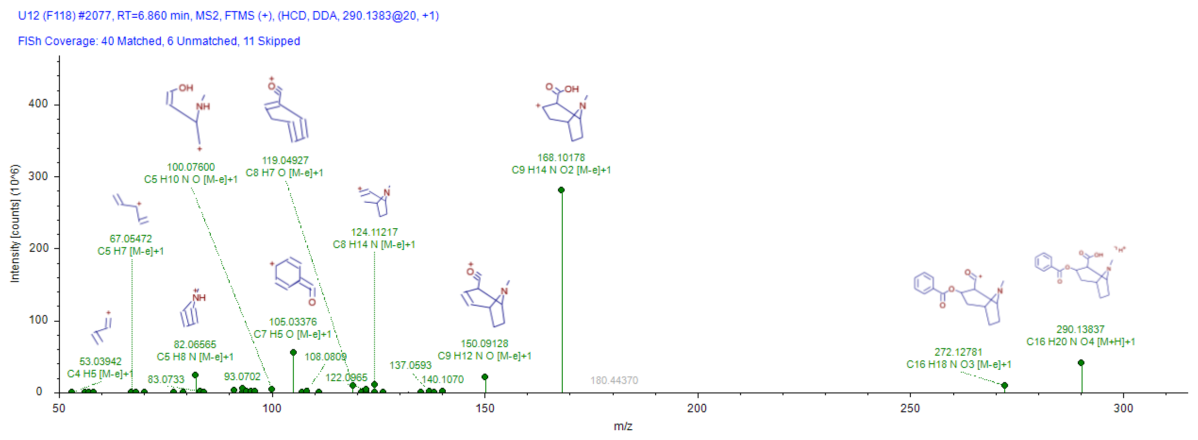

__Figure S 17__ Experimental MS/MS fragmentation spectrum of benzoylecgonine ([M+H]$^+$, m/z 290.1383) detected in urine sample.

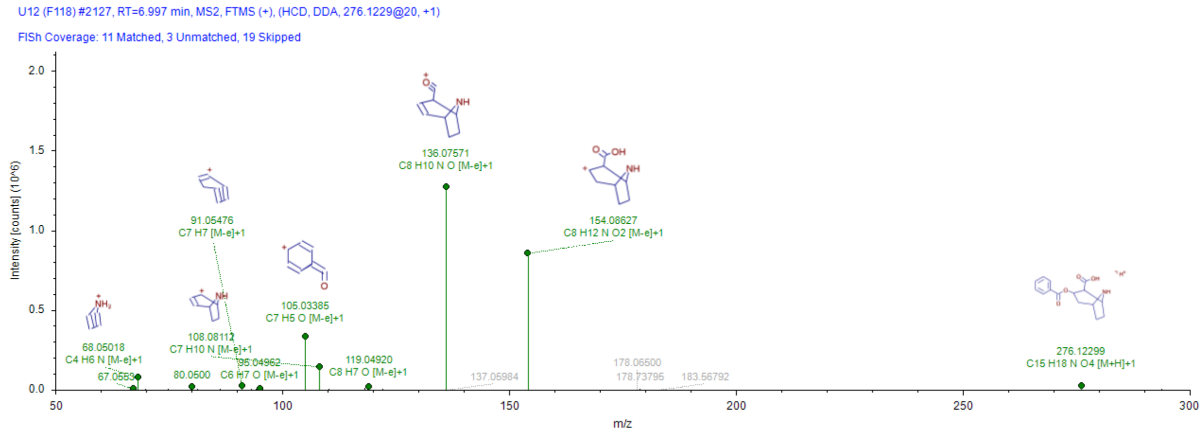

__Figure S 18__ Experimental MS/MS fragmentation spectrum of norbenzoylecgonine ([M+H]$^+$, m/z 276.1229) detected in urine sample.

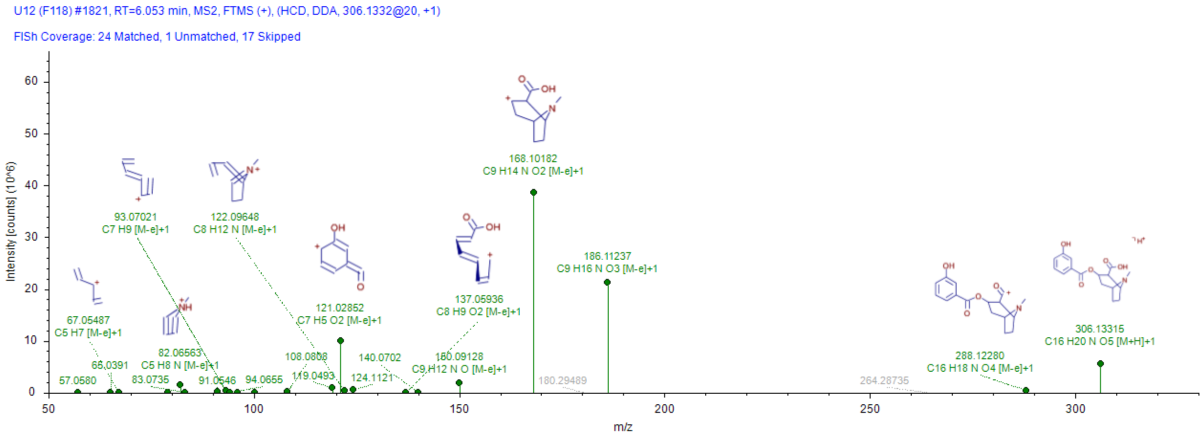

Figure S 19 *Experimental MS/MS fragmentation spectrum of hydroxybenzoylecgonine (isomer I) ([M+H]$^+$, m/z 306.1332) detected in urine sample.*

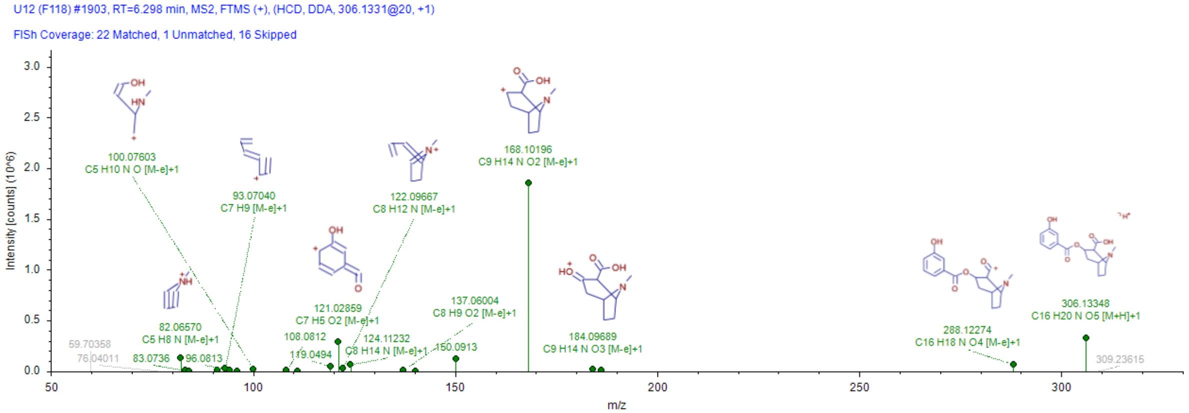

Figure S 20 *Experimental MS/MS fragmentation spectrum of hydroxybenzoylecgonine (isomer II) ([M+H]$^+$, m/z 306.1331) detected in urine sample.*

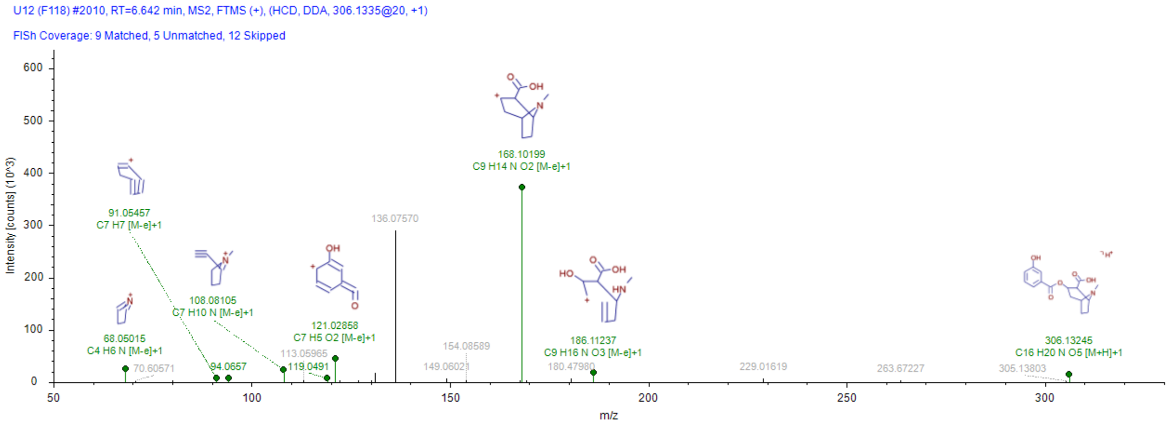

Figure S 21 *Experimental MS/MS fragmentation spectrum of hydroxybenzoylecgonine (isomer III) ($[M+H]^+$, m/z 306.1335) detected in urine sample.*

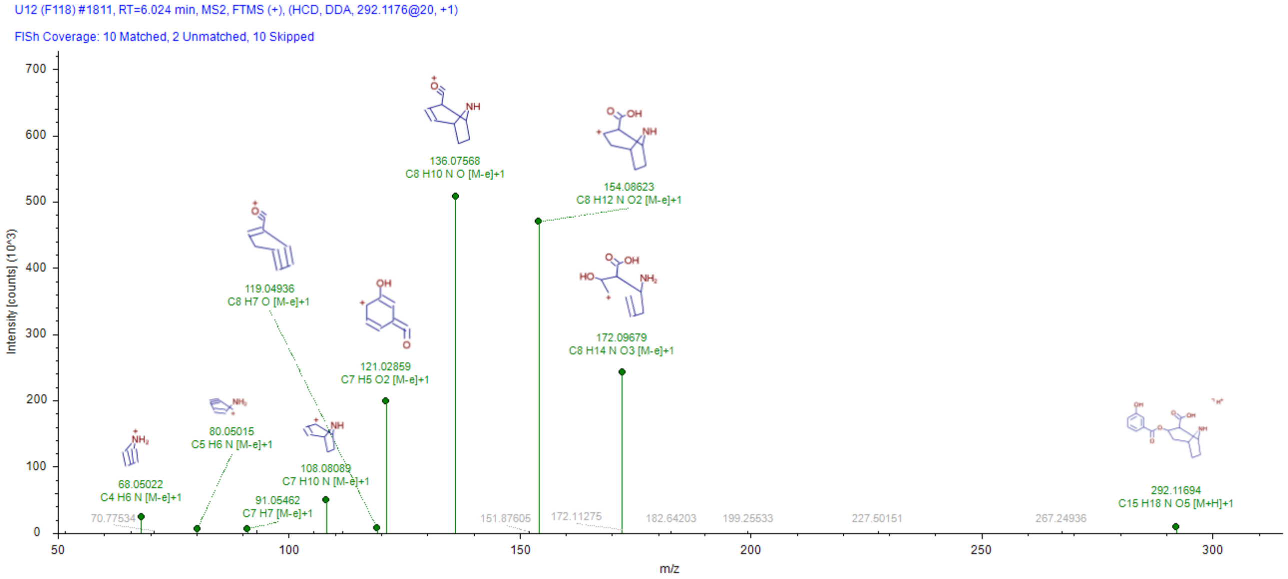

Figure S 22 *Experimental MS/MS fragmentation spectrum of hydroxynor-benzoylecgonine ($[M+H]^+$, m/z 292.1176) detected in urine sample*

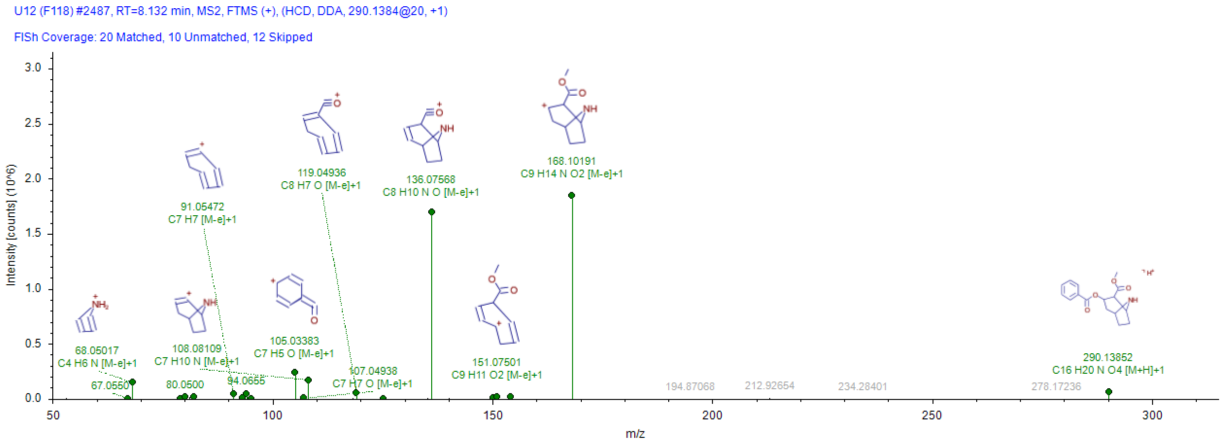

Figure S 23 *Experimental MS/MS fragmentation spectrum of norcocaine ([M+H]⁺, m/z 290.1384) detected in urine sample.*

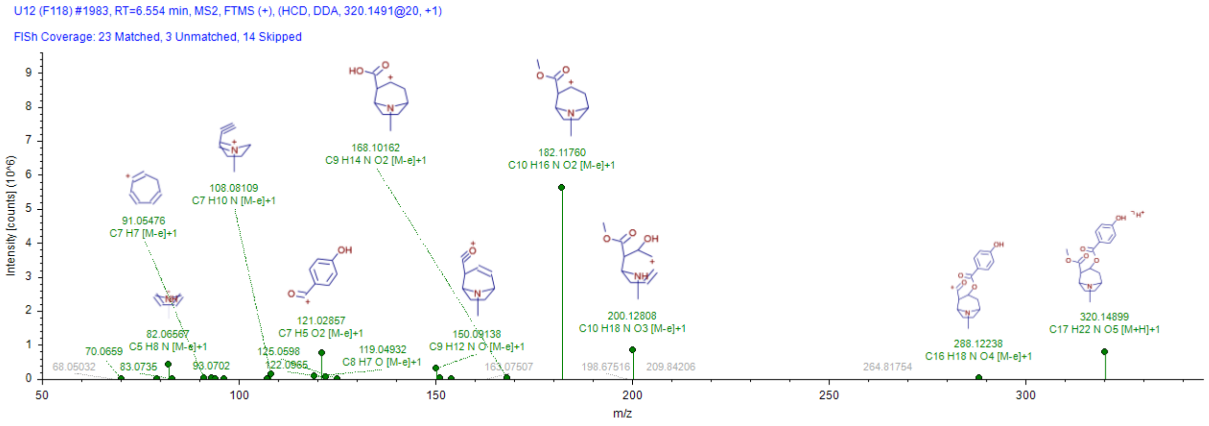

Figure S 24 *Experimental MS/MS fragmentation spectrum of hydroxycocaine (isomer I) ([M+H]⁺, m/z 320.1491) detected in urine sample.*

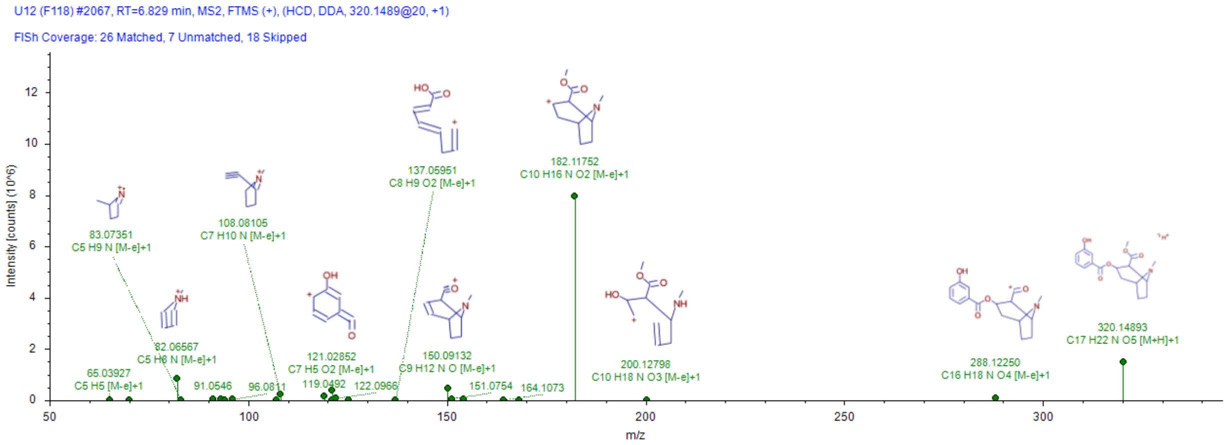

Figure S 25 *Experimental MS/MS fragmentation spectrum of hydroxycocaine (isomer II) ([M+H]⁺, m/z 320.1489) detected in urine sample.*

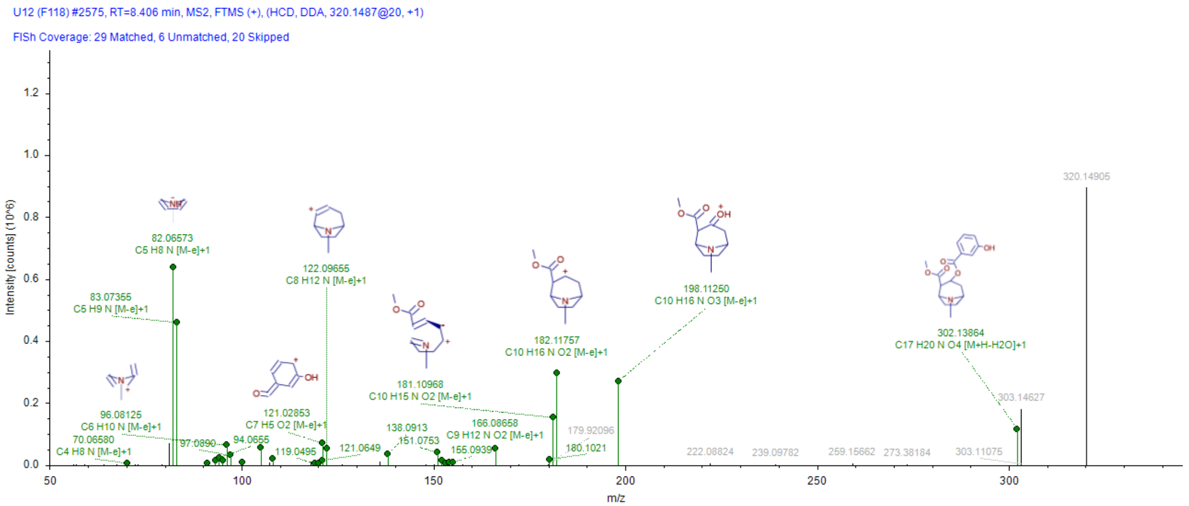

Figure S 26 *Experimental MS/MS fragmentation spectrum of hydroxycocaine (isomer III) ([M+H]⁺, m/z 320.1487) detected in urine sample.*

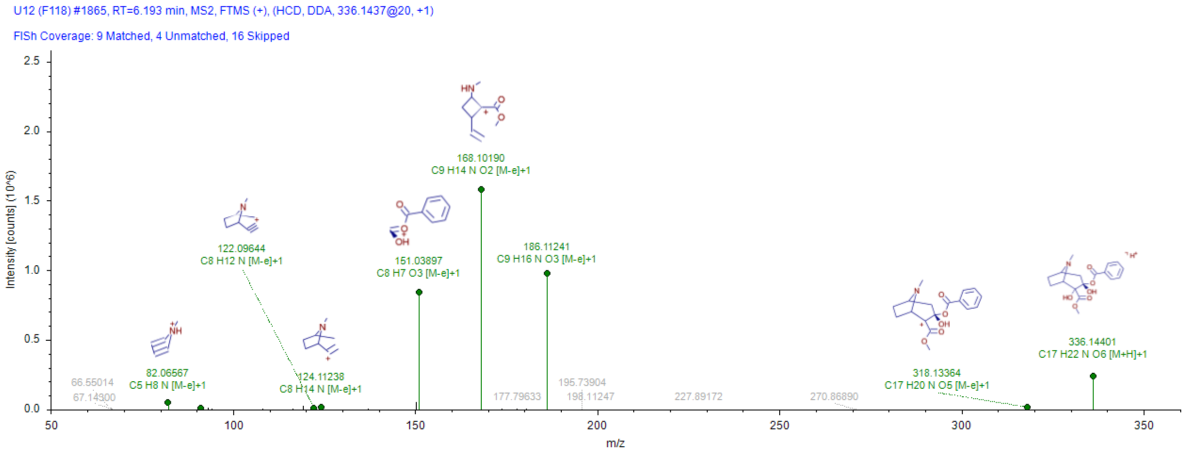

Figure S 27 *Experimental MS/MS fragmentation spectrum of dihydroxycocaine ([M+H]$^{+}$, m/z 336.1437) detected in urine sample.*

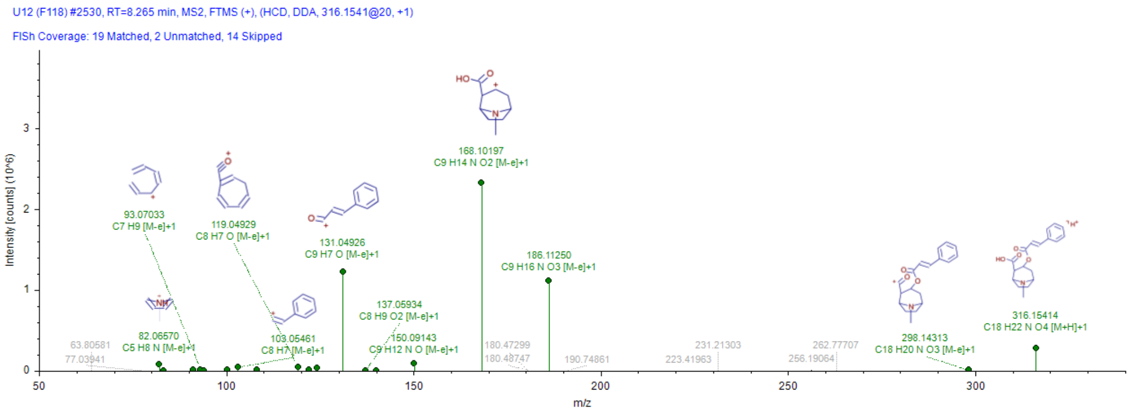

Figure S 28 *Experimental MS/MS fragmentation spectrum of cinnamoylecgonine ([M+H]$^{+}$, m/z 316.1541) detected in urine sample.*

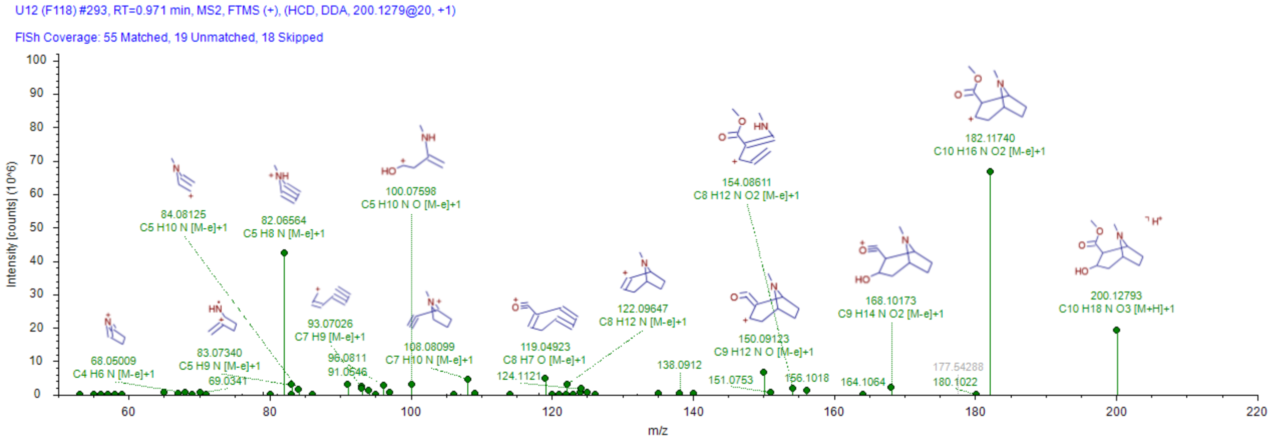

Figure S 29 *Experimental MS/MS fragmentation spectrum of ecgonine methyl ester ([M+H]*$^+$*, m/z 200.1279) detected in urine sample.*

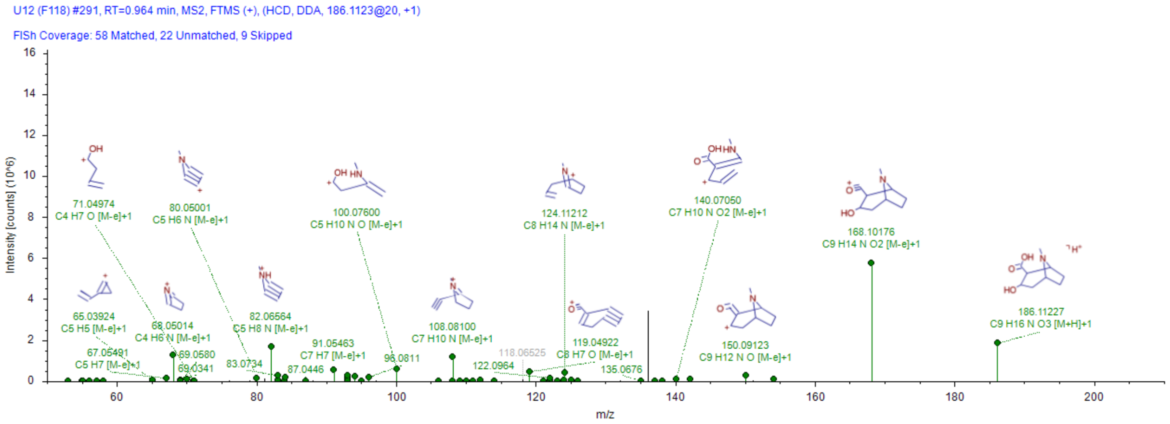

Figure S 30 *Experimental MS/MS fragmentation spectrum of ecgonine ([M+H]*$^+$*, m/z 186.1123) detected in urine sample.*

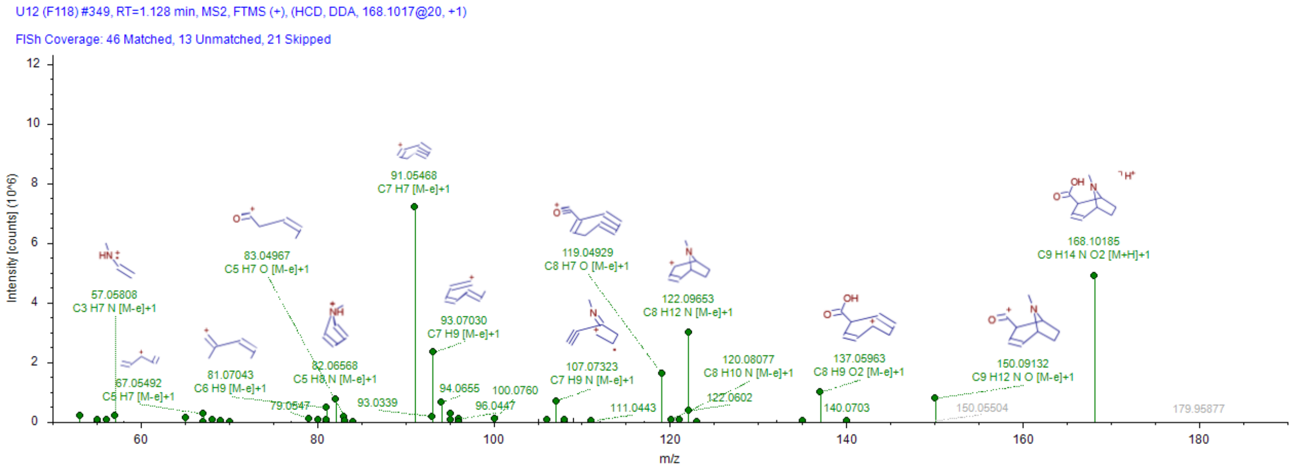

Figure S 31 *Experimental MS/MS fragmentation spectrum of anhydroecgonine ([M+H]⁺, m/z 168.1017) detected in urine sample.*

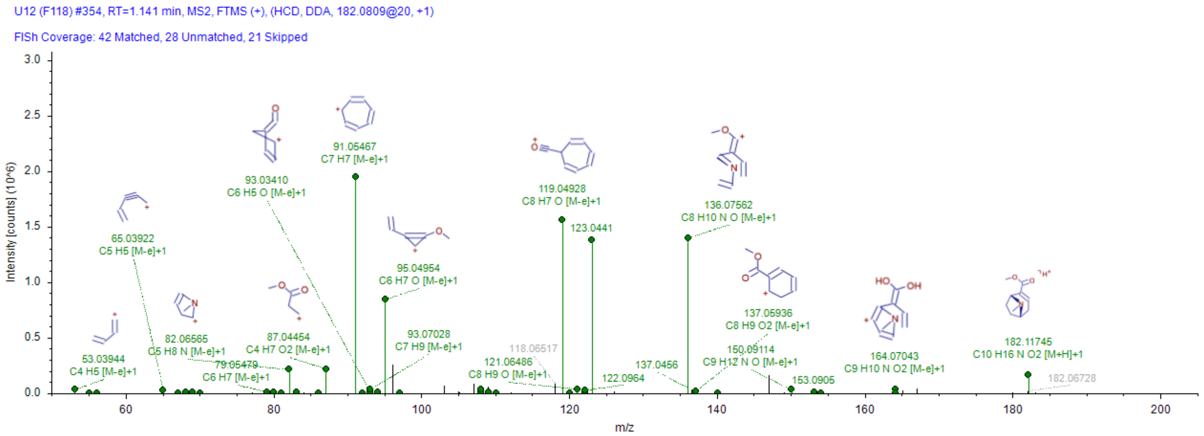

Figure S 32 *Experimental MS/MS fragmentation spectrum of anhydroecgonine methyl ester ([M+H]⁺, m/z 182.0809) detected in urine sample.*

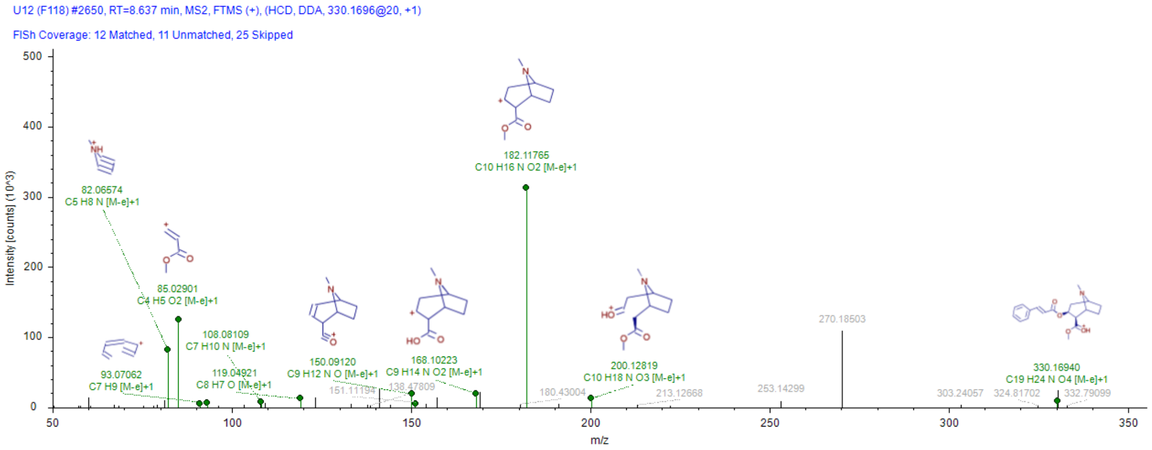

Figure S 33 *Experimental MS/MS fragmentation spectrum of cinnamoylcocaine ([M+H]⁺, m/z 330.1696) detected in urine sample.*

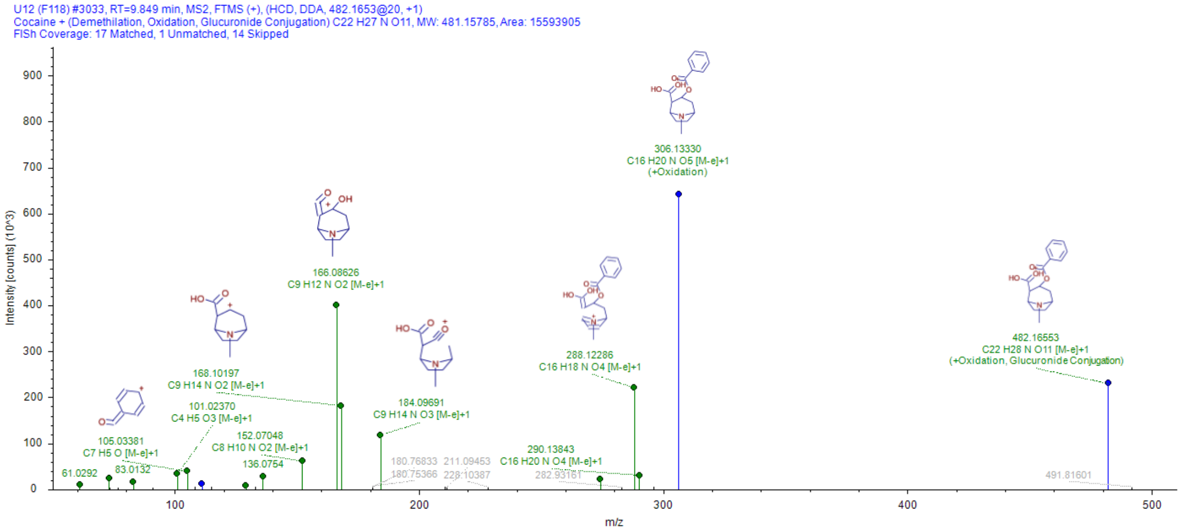

Figure S 34 *Experimental MS/MS fragmentation spectrum of benzoylecgonine-O-glucuronide ([M+H]⁺, m/z 482.1653) detected in urine sample.*

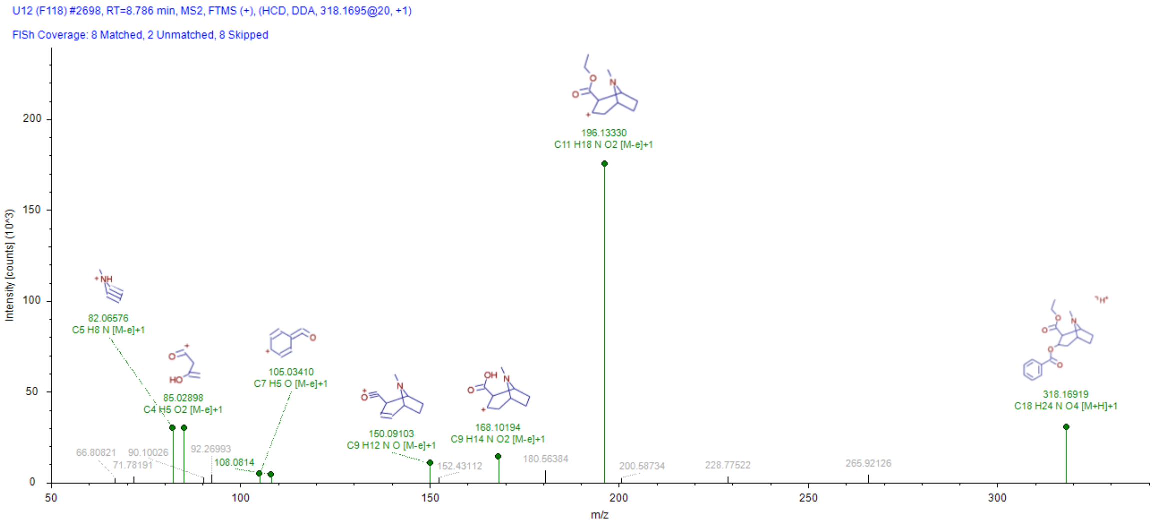

Figure S 35 *Experimental MS/MS fragmentation spectrum of cocaethylene ($[M+H]^+$, m/z 318.1695) detected in urine sample.*

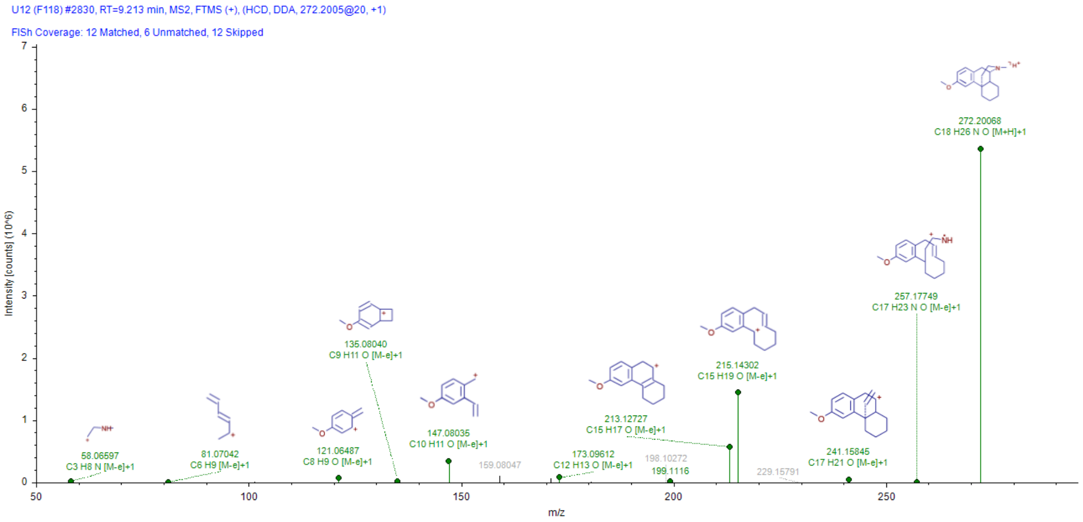

Figure S 36 *Experimental MS/MS fragmentation spectrum of dextromethorphan ($[M+H]^+$, m/z 272.2005) detected in urine sample.*

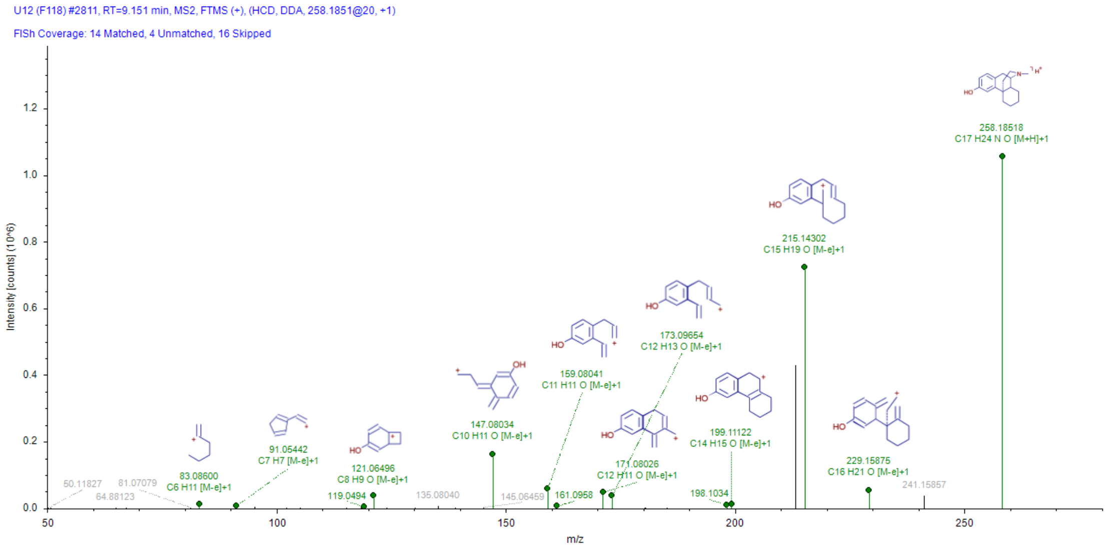

Figure S 37 *Experimental MS/MS fragmentation spectrum of dextrorphan ([M+H]⁺, m/z 258.1851) detected in urine sample.*

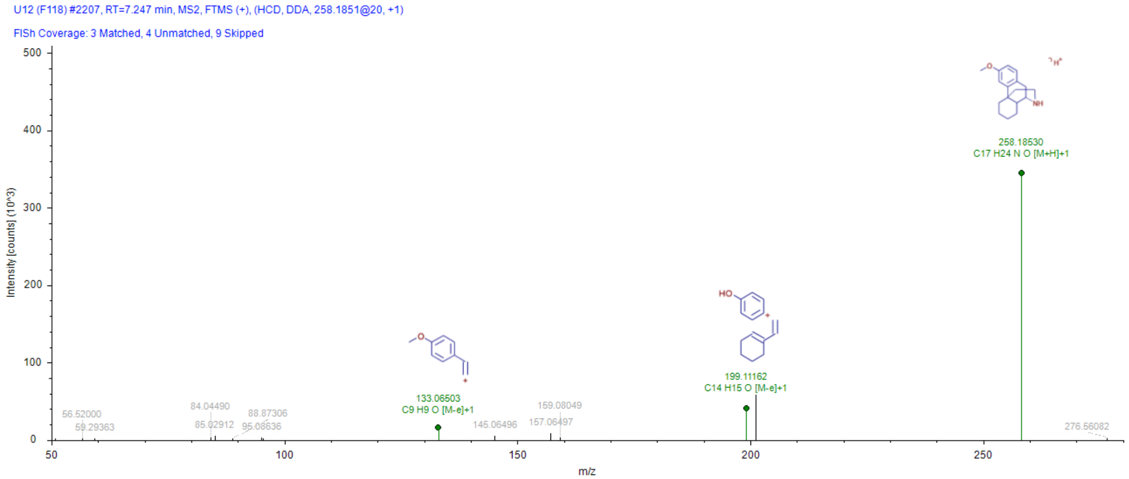

Figure S 38 *Experimental MS/MS fragmentation spectrum of 3-methoxymorphinan ([M+H]⁺, m/z 258.1851) detected in urine sample.*

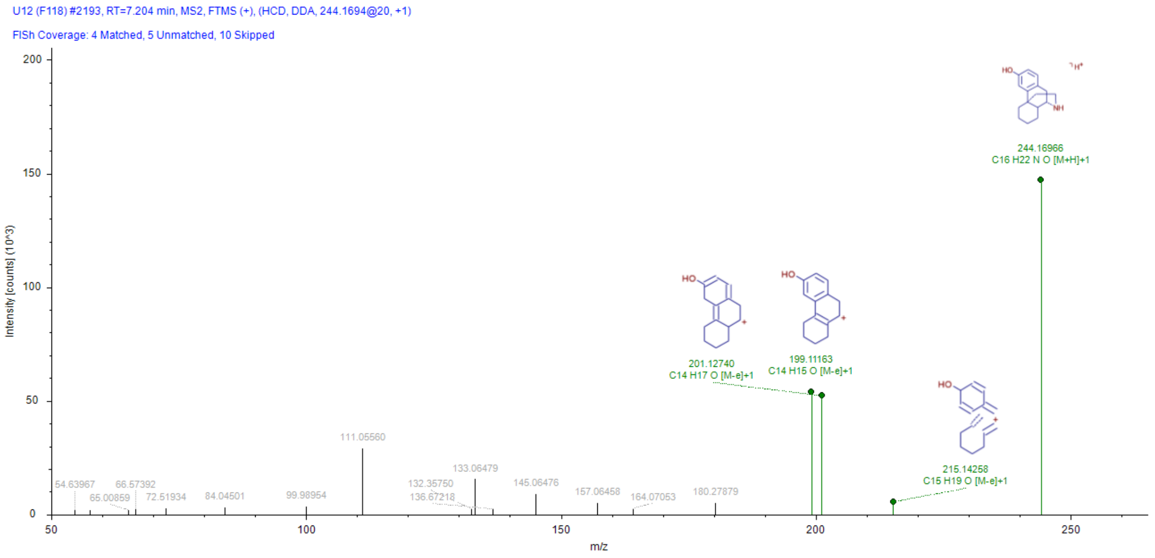

Figure S 39 *Experimental MS/MS fragmentation spectrum of 3-hydroxymorphinan ([M+H]$^+$, m/z 244.1694) detected in urine sample.*

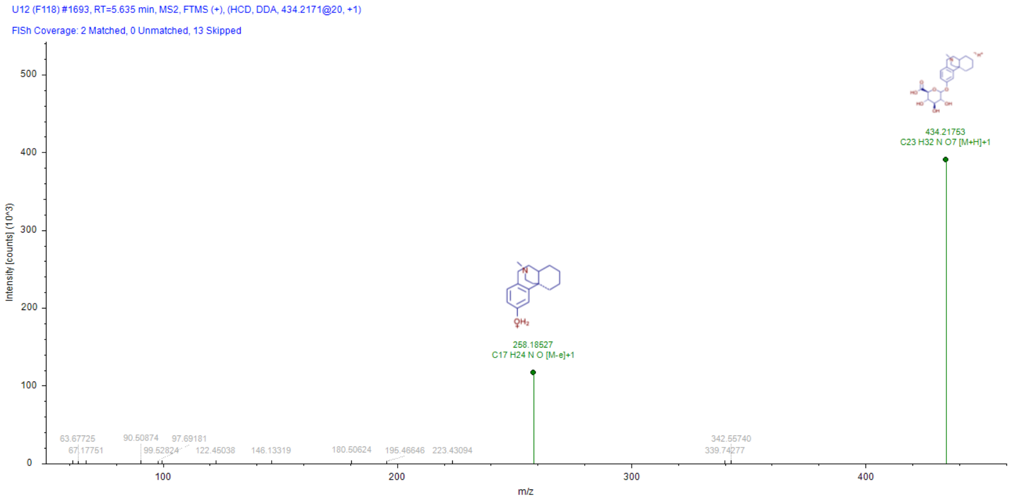

Figure S 40 *Experimental MS/MS fragmentation spectrum of dextrorphan-O-glucuronide ([M+H]$^+$, m/z 434.2171) detected in urine sample.*

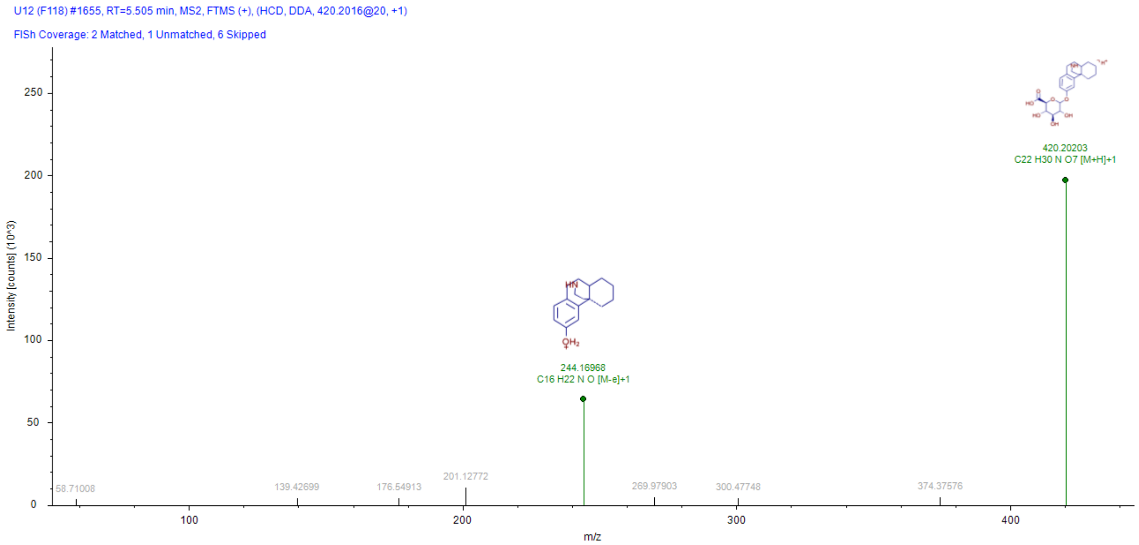

Figure S 41 *Experimental MS/MS fragmentation spectrum of 3-hydroxymorphinan-O-glucuronide ([M+H]$^{+}$, m/z 420.2016) detected in urine sample.*

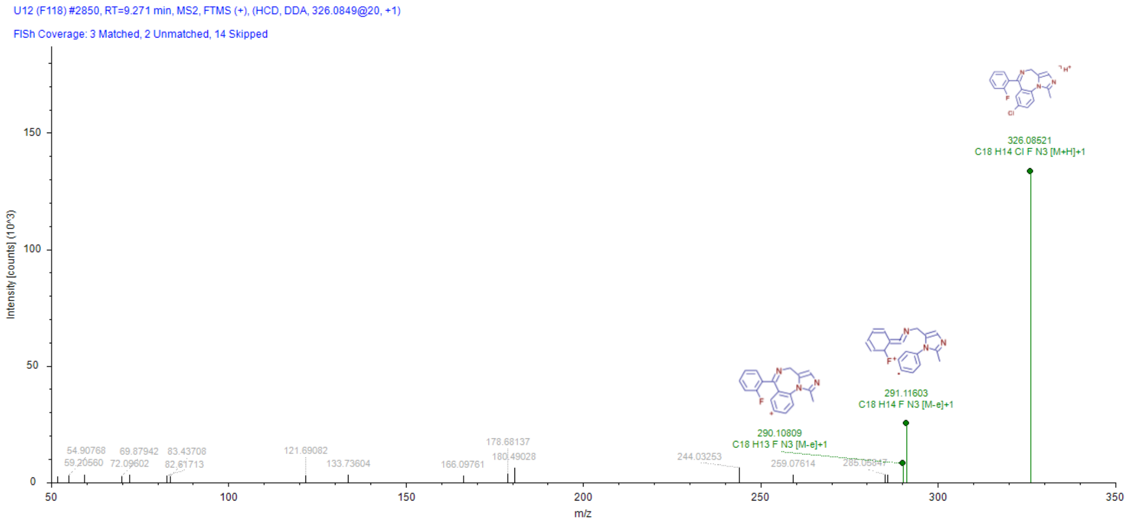

Figure S 42 *Experimental MS/MS fragmentation spectrum of midazolam ([M+H]$^{+}$, m/z 326.0849) detected in urine sample.*

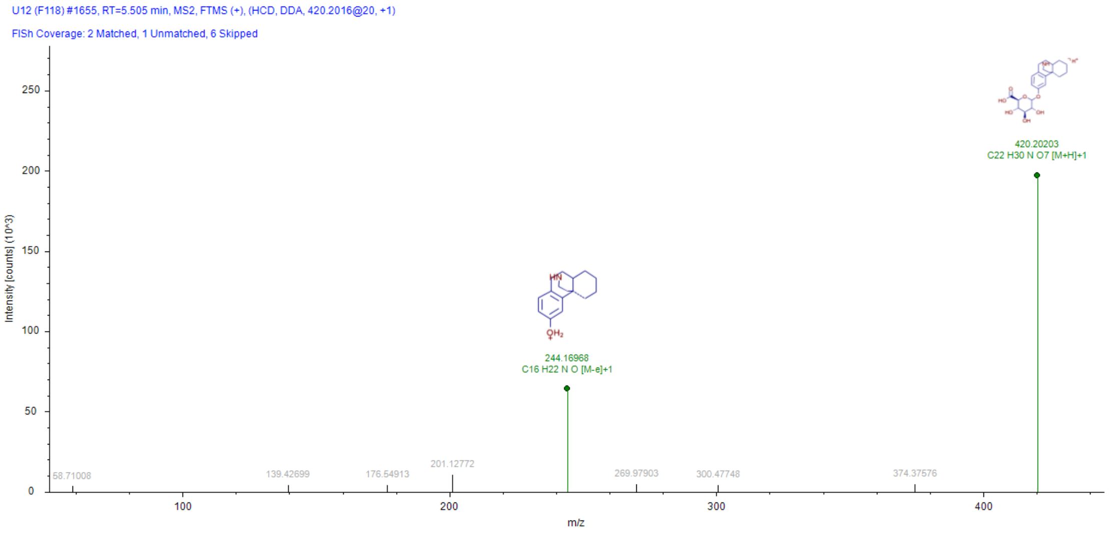

__Figure S 43__ Experimental MS/MS fragmentation spectrum of hydroxymidazolam-O-glucuronide ([M+H]$^{+}$, m/z 420.2016) detected in urine sample.

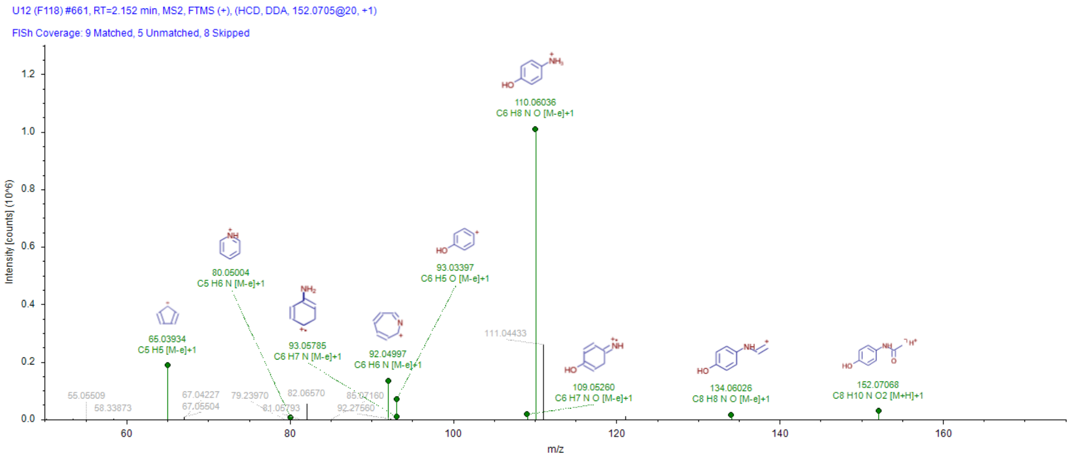

__Figure S 44__ Experimental MS/MS fragmentation spectrum of acetaminophen ([M+H]$^{+}$, m/z 152.0705) detected in urine sample.

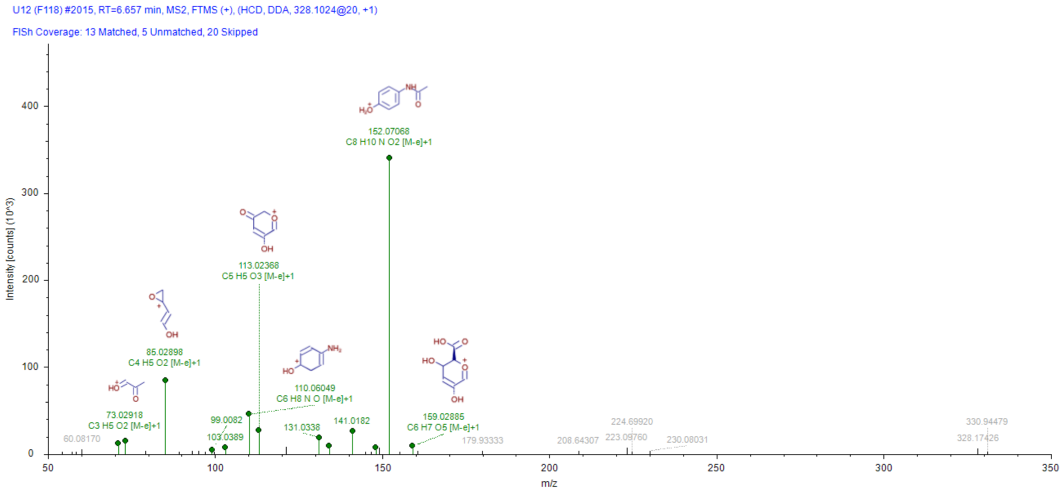

Figure S 45 *Experimental MS/MS fragmentation spectrum of acetaminophen-O-glucuronide ([M+H]*$^+$*, m/z 328.1024) detected in urine sample.*

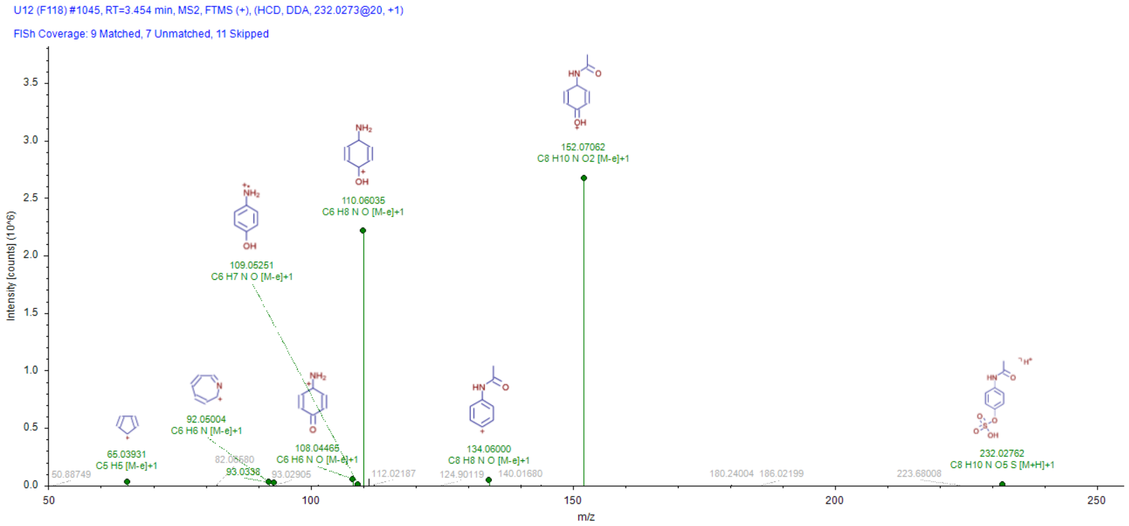

Figure S 46 *Experimental MS/MS fragmentation spectrum of acetaminophen sulfate ([M+H]*$^+$*, m/z 232.0273) detected in urine sample.*

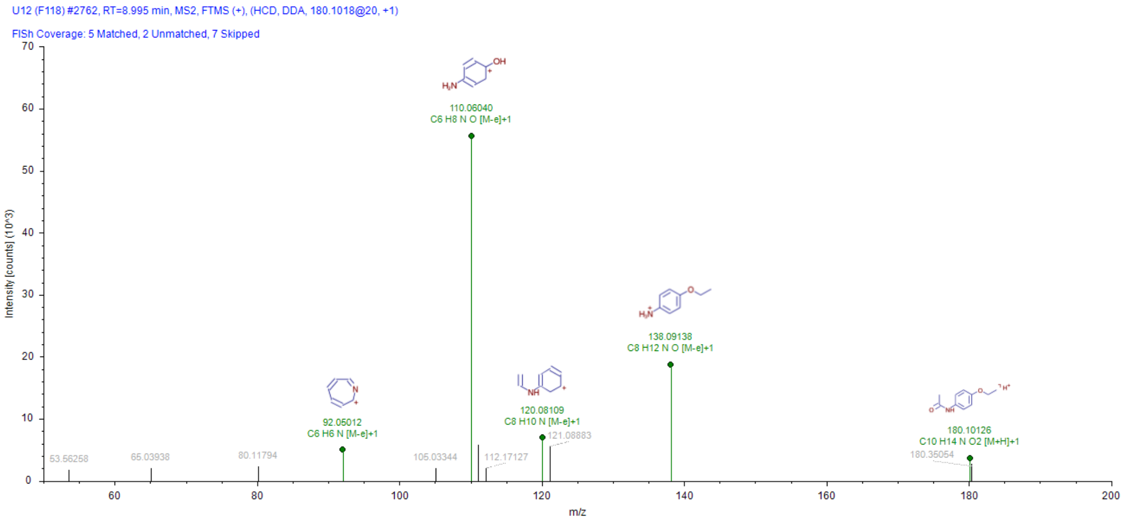

__Figure S 47__ Experimental MS/MS fragmentation spectrum of phenacetin ([M+H]$^+$, m/z 180.1018) detected in urine sample.

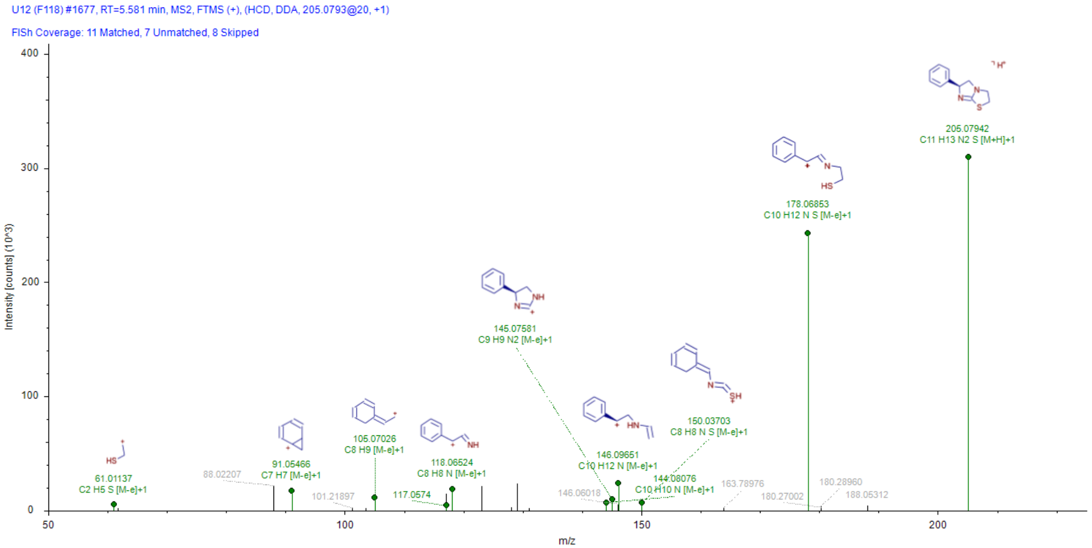

__Figure S 48__ Experimental MS/MS fragmentation spectrum of levamisole ([M+H]$^+$, m/z 205.0793) detected in urine sample.

***Table S** 7 Overview of all MetID-proposed metabolites, including their predicted structures and associated phase I/II metabolic transformations.*
Abbreviations: 3-OH-morphinan, 3-hydroxymorphinan; 3-OH-morphinan-O-Glu, 3-hydroxymorphinan-O-glucuronide; APAP, acetaminophen (paracetamol); APAP-O-Glu, acetaminophen-O-glucuronide; APAP sulfate, acetaminophen sulfate; BZE, benzoylecgonine; CEC, cinnamoylecgonine; COC, cocaine; diOH-COC, dihydroxycocaine; DXM, dextromethorphan; DXO, dextrorphan; DXO-O-Glu, dextrorphan-O-glucuronide; MDZ, midazolam; NBZE, norbenzoylecgonine; NCOC, norcocaine; OH-BZE (I), hydroxybenzoylecgonine (isomer I); OH-BZE (II), hydroxybenzoylecgonine (isomer II); OH-COC (I), hydroxycocaine (isomer I); OH-COC (II), hydroxycocaine (isomer II); OH-COC (III), hydroxycocaine (isomer III); OH-MDZ-O-Glu, hydroxymidazolam-O-glucuronide; OH-NBZE, hydroxynorbenzoylecgonine.

ID	Matrix	Parent	Metabolite (Putative)	Composition	Transformation(s)	ΔFormula (vs Parent)	ΔMass (ppm)	Exact Mass	m/z $[M+H]^+$	RT [min]	Max Peak Area (a.u.)	Confidence Level
1	Blood/urine	COC	BZE	C16 H19 N O4	Demethilation	-(C H2)	-1.52	289.1310	290.1382	6.88	4.54E+09	1
2	Blood/urine	COC	NBZE	C15 H17 N O4	Demethilation, Demethilation	-(C2 H4)	-1.06	275.1155	276.1227	7.03	5.46E+08	2a
3	Blood/urine	COC	OH-BZE (isomer I)	C16 H19 N O5	Demethilation, Oxidation	-(C H2) +(O)	-1.28	305.1259	306.1332	6.04	4.09E+08	2b
4	Blood/urine	COC	OH-BZE (isomer II)	C16 H19 N O5	Demethilation, Oxidation	-(C H2) +(O)	-1.28	305.1259	306.1332	6.33	9.58E+07	2b
5	Blood/urine	COC	OH-NBZE	C15 H17 N O5	Demethilation, Demethilation, Oxidation	-(C2 H4) +(O)	-0.73	291.1105	292.1177	7.82	2.66E+08	2b
6	Blood/urine	COC	OH-COC (isomer I)	C17 H21 N O5	Oxidation	+(O)	-0.73	319.1417	320.149	6.58	8.46E+07	2b
7	Urine	COC	NCOC	C16 H19 N O4	Demethilation	-(C H2)	-1.21	289.1311	290.1383	8.16	2.28E+08	2a
8	Urine	COC	OH-COC (isomer II)	C17 H21 N O5	Oxidation	+(O)	-1.4	319.1415	320.1488	6.83	2.98E+07	2b
9	Urine	COC	OH-COC (isomer III)	C17 H21 N O5	Oxidation	+(O)	-1.49	319.1415	320.1488	8.42	2.27E+07	2b
10	Urine	COC	CEC	C18 H21 N O4	Desaturation, Methylation	+(C)	-0.80	315.1468	316.1541	8.27	1.80E+07	2b
11	Urine	COC	diOH-COC	C17 H21 N O6	Oxidation, Oxidation	+(O2)	-1.64	335.1363	336.1436	6.20	1.72E+07	3
12	Urine	COC	OH-BZE (isomer III)	C16 H19 N O5	Demethilation, Oxidation	-(C H2) +(O)	-0.68	305.1261	306.1334	6.66	7.22E+06	2b
13	Blood/urine	DXM	DXO	C17 H23 N O	Demethilation	-(C H2)	-0.95	257.1777	258.185	9.17	4.26E+07	2a
14	Urine	DXM	3-methoxymorphinan	C17 H23 N O	Demethilation	-(C H2)	-0.71	257.1778	258.1851	7.25	1.94E+06	3
15	Urine	DXM	3-OH-morphinan	C16 H21 N O	Demethilation, Demethilation	-(C2 H4)	-0.71	243.1621	244.1694	7.19	1.70E+06	2b
16	Urine	DXM	DXO-O-Glu	C23 H31 N O7	Demethilation, Glucuronide Conjugation	+(C5 H6 O6)	-0.31	433.2099	434.2172	5.55	2.66E+06	2b
17	Urine	DXM	3-OH-morphinan-O-Glu	C22 H29 N O7	Demethilation, Demethilation, Glucuronide Conjugation	+(C4 H4 O6)	-0.26	419.1943	420.2016	5.49	1.25E+06	2b
18	Urine	MDZ	OH-MDZ-O-Glu	C24 H21 Cl F N3 O7	Oxidation, Glucuronide Conjugation	+(C6 H8 O7)	-0.46	517.1050	518.1122	8.34	1.07E+07	2b
19	Urine	APAP	APAP-O-Glu	C14 H17 N O8	Glucuronide Conjugation	+(C6 H8 O6)	-0.75	327.0950	328.1025	6.65	2.33E+06	2b
20	Urine	APAP	APAP sulfate	C8 H9 N O5 S	Sulfation	+(O3 S)	-0.92	231.0199	232.0272	3.42	1.23E+08	2b

Table S 8 List of parent compounds and associated blood metabolites identified within the transformation-aware molecular networking workflow. *For each compound, the matrix, parent compound (seed), putative assignment, elemental composition, mass error (ΔMass, ppm), exact mass, measured m/z, reference ion, retention time (RT, min), and peak area (a.u.) are reported. Identification confidence levels were assigned according to the Schymanski framework (Section 2.9). The table provides a comprehensive overview of compound-level analytical parameters supporting structural annotation of blood metabolites and corresponding parent drugs.*

Abbreviations: AEC, anhydroecgonine; APAP, acetaminophen (paracetamol); BZE, benzoylecgonine; COC, cocaine; DXM, dextromethorphan; DXO, dextrorphan; EC, ecgonine; EME, ecgonine methyl ester; LEV, levamisole; MDZ, midazolam; NBZE, norbenzoylecgonine; OH-BZE (I), hydroxybenzoylecgonine (isomer I); OH-BZE (II), hydroxybenzoylecgonine (isomer II); OH-COC (I), hydroxycocaine (isomer I); OH-NBZE, hydroxynorbenzoylecgonine.

ID	Matrix	Parent	Putative Assignment	Composition	ΔMass (ppm)	Exact Mass	m/z	Reference Ion	RT [min]	Peak Area (a.u.)
1	Blood	COC	COC	C17 H21 N O4	0.28	303.1471	304.1544	[M+H]+1	8.01	1.35E+06
2	Blood	COC	BZE	C16 H19 N O4	-0.57	289.1312	290.1385	[M+H]+1	6.91	4.79E+07
3	Blood	COC	NBZE	C15 H17 N O4	0.71	275.1160	276.1232	[M+H]+1	7.03	2.29E+06
4	Blood	COC	OH-BZE (isomer I)	C16 H19 N O5	-0.18	305.1263	306.1335	[M+H]+1	6.02	1.44E+06
5	Blood	COC	OH-BZE (isomer II)	C16 H19 N O5	0.02	305.1263	306.1336	[M+H]+1	6.32	2.19E+05
6	Blood	COC	OH-NBZE	C15 H17 N O5	0.00	291.1107	292.1180	[M+H]+1	7.82	1.46E+05
7	Blood	COC	OH-COC (isomer I)	C17 H21 N O5	0.71	319.1422	320.1495	[M+H]+1	6.55	1.26E+05
8	Blood	COC	EME	C10 H17 N O3	0.65	199.1210	200.1283	[M+H]+1	0.98	5.29E+06
9	Blood	COC	EC	C9 H15 N O3	0.76	185.1053	186.1126	[M+H]+1	0.96	2.20E+06
10	Blood	COC	AEC	C9 H13 N O2	-0.22	167.0946	168.1019	[M+H]+1	1.13	9.07E+05
11	Blood	DXM	DXM	C18 H25 N O	0.47	271.1937	272.2010	[M+H]+1	9.25	5.81E+05
12	Blood	DXM	DXO	C17 H23 N O	1.07	257.1782	258.1855	[M+H]+1	9.18	2.26E+05
13	Blood	MDZ	MDZ	C18 H13 Cl F N3	0.56	325.0784	326.0857	[M+H]+1	9.26	3.55E+06

Table S 9 List of network-derived connections for blood metabolites identified within the transformation-aware molecular network. *For each node, the matrix, seed node, putative assignment, elemental composition, formula change relative to the parent compound (ΔFormula), linked node(s), edge direction(s), MS^n score (%), spectral coverage (%), number of matched fragments, and confidence level are reported. Connections were established based on combined MS/MS spectral similarity and curated biotransformation rules implemented in the Molecular Networking workflow (Compound Discoverer 3.4). Only features fulfilling the predefined similarity and quality thresholds were retained. The table summarizes the relational metrics supporting node connectivity within the blood molecular network.*

Abbreviations: 3-OH-morphinan, 3-hydroxymorphinan; 3-OH-morphinan-O-Glu, 3-hydroxymorphinan-O-glucuronide; APAP, acetaminophen (paracetamol); APAP-O-Glu, acetaminophen-O-glucuronide; APAP sulfate, acetaminophen sulfate; BZE, benzoylecgonine; CEC, cinnamoylecgonine; COC, cocaine; diOH-COC, dihydroxycocaine; DXM, dextromethorphan; DXO, dextrorphan; DXO-O-Glu, dextrorphan-O-glucuronide; MDZ, midazolam; NBZE, norbenzoylecgonine; NCOC, norcocaine; OH-BZE (I), hydroxybenzoylecgonine (isomer I); OH-BZE (II), hydroxybenzoylecgonine (isomer II); OH-COC (I), hydroxycocaine (isomer I); OH-COC (II), hydroxycocaine (isomer II); OH-COC (III), hydroxycocaine (isomer III); OH-MDZ-O-Glu, hydroxymidazolam-O-glucuronide; OH-NBZE, hydroxynorbenzoylecgonine.

ID	Matrix	Seed Node	Putative Assignment	Elemental Composition	ΔFormula (vs Parent)	Linked to	Direction(s)	MSn Score (%)	Coverage (%)	Matched Fragments	Confidence Level
1	Blood	COC	COC	C17 H21 N O4	-	COC seed	Fwd	51	43/60	3/3	1
2	Blood	COC	BZE	C16 H19 N O4	-(C H2)	COC	Fwd	37	60/14	3/3	1
3	Blood	COC	NBZE	C15 H17 N O4	-(C2 H4)	BZE	Fwd	53	23/83	5/5	2a
4	Blood	COC	OH-BZE (isomer I)	C16 H19 N O5	-(C H2) +(O)	COC seed, BZE, OH-COC	Fwd	51;37;60	43-14/60; 100/20	3/3; 1/1	2b
5	Blood	COC	OH-BZE (isomer II)	C16 H19 N O5	-(C H2) +(O)	COC seed, BZE, OH-COC, OH-BZE (isomer I)	Fwd	48; 38; 67; 53	29-9-40/67; 100/33	2/2;1/1	2b
6	Blood	COC	OH-NBZE	C15 H17 N O5	-(C2 H4) +(O)	BZE, NBZE	Fwd	52; 58	5-17/100	1/1	2b
7	Blood	COC	OH-COC (isomer I)	C17 H21 N O5	+(O)	COC seed, COC	Fwd	52; 58	14-20/100	1/1	2b
8	Blood	COC	EME	C10 H17 N O3	-(C7 H4 O)	COC seed	Fwd	44	71/16	5/5	2a
9	Blood	COC	EC	C9 H15 N O3	-(C8 H6 O)	EME	Fwd	66	52/81	16/17	2a
10	Blood	COC	AEC	C9 H13 N O2	-(C8 H8 O2)	EME; EC	Fwd	55; 57	10-14/100	3/3	2a
11	Blood	DXM	DXM	C18 H25 N O	-	DXM seed	Fwd	56	11/100	2/2	2a
12	Blood	DXM	DXO	C17 H23 N O	-(C H2)	DXM	Fwd	75	100/50	2/2	2a
13	Blood	MDZ	MDZ	C18 H13 Cl F N3	-	MDZ seed	Fwd	50	40/60	2/3	1

Table S 10 List of parent compounds and associated urine metabolites identified within the transformation-aware molecular networking workflow. *For each compound, the matrix, parent compound (seed), putative assignment, elemental composition, mass error (ΔMass, ppm), exact mass, measured m/z, reference ion, retention time (RT, min), and peak area (a.u.) are reported. Identification confidence levels were assigned according to the Schymanski framework (Section 2.9). The table provides a comprehensive overview of compound-level analytical parameters supporting structural annotation of urine metabolites and corresponding parent drugs.*

Abbreviations: 3-OH-morphinan, 3-hydroxymorphinan; 3-OH-morphinan-O-Glu, 3-hydroxymorphinan-O-glucuronide; AEC, anhydroecgonine; AEME, anhydroecgonine methyl ester; APAP, acetaminophen (paracetamol); APAP-O-Glu, acetaminophen-O-glucuronide; APAP sulfate, acetaminophen sulfate; BZE, benzoylecgonine; BZE-O-Glu, benzoylecgonine-O-glucuronide; CEC, cinnamoylecgonine; CCOC, cinnamoylcocaine; CE, cocaethylene; COC, cocaine; diOH-COC, dihydroxycocaine; DXM, dextromethorphan; DXO, dextrorphan; DXO-O-Glu, dextrorphan-O-glucuronide; EC, ecgonine; EME, ecgonine methyl ester; LEV, levamisole; MDZ, midazolam; NBZE, norbenzoylecgonine; NCOC, norcocaine; OH-BZE (I), hydroxybenzoylecgonine (isomer I); OH-

BZE (II), hydroxybenzoylecgonine (isomer II); OH-BZE (III), hydroxybenzoylecgonine (isomer III); OH-COC (I), hydroxycocaine (isomer I); OH-COC (II), hydroxycocaine (isomer II); OH-COC (III), hydroxycocaine (isomer III); OH-MDZ-O-Glu, hydroxymidazolam-O-glucuronide; OH-NBZE, hydroxynorbenzoylecgonine; PHE, phenacetin.

ID	Matrix	Parent	Putative Assignment	Composition	ΔMass (ppm)	Exact Mass	m/z	Reference Ion	RT [min]	Peak Area (a.u.)
1	Urine	COC	COC	C17 H21 N O4	-1.33	303.1467	304.1539	[M+H]+1	8.01	2.31E+09
2	Urine	COC	BZE	C16 H19 N O4	-1.52	289.1310	290.1382	[M+H]+1	6.88	4.54E+09
3	Urine	COC	NBZE	C15 H17 N O4	-1.06	275.1155	276.1227	[M+H]+1	7.03	5.46E+08
4	Urine	COC	OH-BZE (isomer I)	C16 H19 N O5	-1.28	305.1259	306.1332	[M+H]+1	6.04	4.09E+08
5	Urine	COC	OH-BZE (isomer II)	C16 H19 N O5	-1.28	305.1259	306.1332	[M+H]+1	6.33	9.58E+07
6	Urine	COC	OH-BZE (isomer III)	C16 H19 N O5	-0.68	305.1261	306.1334	[M+H]+1	6.66	7.22E+06
7	Urine	COC	OH-NBZE	C15 H17 N O5	-0.73	291.1105	292.1177	[M+H]+1	7.82	1.50E+07
8	Urine	COC	NCOC	C16 H19 N O4	-1.21	289.1311	290.1383	[M+H]+1	8.16	2.28E+08
9	Urine	COC	OH-COC (isomer I)	C17 H21 N O5	-0.73	319.1417	320.1490	[M+H]+1	6.58	8.46E+07
10	Urine	COC	OH-COC (isomer II)	C17 H21 N O5	-1.40	319.1415	320.1488	[M+H]+1	6.83	2.98E+07
11	Urine	COC	OH-COC (isomer III)	C17 H21 N O5	-1.49	319.1415	320.1488	[M+H]+1	8.42	2.27E+07
12	Urine	COC	diOH-COC	C17 H21 N O6	-1.64	335.1363	336.1436	[M+H]+1	6.20	1.72E+07
13	Urine	COC	CEC	C18 H21 N O4	-0.80	315.1468	316.1541	[M+H]+1	8.27	1.80E+07
14	Urine	COC	EME	C10 H17 N O3	-1.42	199.1206	200.1278	[M+H]+1	0.98	9.60E+08
15	Urine	COC	EC	C9 H15 N O3	-0.81	185.1050	186.1123	[M+H]+1	0.97	9.69E+07
16	Urine	COC	AEC	C9 H13 N O2	-1.50	167.0944	168.1017	[M+H]+1	1.12	1.61E+08
17	Urine	COC	AEME	C10 H15 N O2	-0.35	181.1102	182.1175	[M+H]+1	2.11	7.15E+07
18	Urine	COC	CCOC	C19 H23 N O4	-1.03	329.1624	330.1697	[M+H]+1	8.62	2.50E+06
19	Urine	COC	BZE-O-Glu	C22 H27 N O11	-1.16	481.1579	482.1651	[M+H]+1	9.87	1.56E+07
20	Urine	COC	CE	C18 H23 N O4	-1.55	317.1622	318.1695	[M+H]+1	8.77	1.42E+06
21	Urine	DXM	DXM	C18 H25 N O	-1.55	271.1932	272.2005	[M+H]+1	9.24	1.91E+08
22	Urine	DXM	DXO	C17 H23 N O	-0.95	257.1777	258.1850	[M+H]+1	9.17	4.26E+07
23	Urine	DXM	3-methoxymorphinan	C17 H23 N O	-0.71	257.1778	258.1851	[M+H]+1	7.25	1.94E+06

24	Urine	DXM	3-OH-morphinan	C16 H21 N O	-0.71	243.1621	244.1694	[M+H]+1	7.19	1.70E+06
25	Urine	DXM	DXO-O-Glu	C23 H31 N O7	-0.52	433.2098	434.2171	[M+H]+1	5.55	2.66E+06
26	Urine	DXM	3-OH-morphinan-O-Glu	C22 H29 N O7	-0.26	419.1943	420.2016	[M+H]+1	5.49	1.25E+06
27	Urine	MDZ	MDZ	C18 H13 Cl F N3	-1.88	325.0776	326.0849	[M+H]+1	9.26	1.06E+06
28	Urine	MDZ	OH-MDZ-O-Glu	C24 H21 Cl F N3 O7	-0.46	517.1050	518.1122	[M+H]+1	8.34	1.07E+07
29	Urine	APAP	APAP	C8 H9 N O2	-0.83	151.0632	152.0705	[M+H]+1	2.16	5.46E+07
30	Urine	APAP	APAP-O-Glu	C14 H17 N O8	-0.75	327.0952	328.1025	[M+H]+1	6.65	2.33E+06
31	Urine	APAP	APAP sulfate	C8 H9 N O5 S	-0.92	231.0199	232.0272	[M+H]+1	3.42	1.23E+08
32	Urine	APAP	PHE	C10 H13 N O2	-0.55	179.0945	180.1018	[M+H]+1	8.97	1.45E+06
33	Urine	LEV	LEV	C11 H12 N2 S	-0.39	204.0720	205.0793	[M+H]+1	5.58	7.54E+06

Table S 11 List of network-derived connections for urine metabolites identified within the transformation-aware molecular network. *For each node, the matrix, seed node, putative assignment, elemental composition, formula change relative to the parent compound (ΔFormula), linked node(s), edge direction(s), MS^n score (%), spectral coverage (%), number of matched fragments, and confidence level are reported. Connections were established based on combined MS/MS spectral similarity and curated biotransformation rules implemented in the Molecular Networking workflow (Compound Discoverer 3.4). Only features fulfilling the predefined similarity and quality thresholds were retained. The table summarizes the relational metrics supporting node connectivity within the urine molecular network.*

Abbreviations: 3-OH-morphinan, 3-hydroxymorphinan; 3-OH-morphinan-O-Glu, 3-hydroxymorphinan-O-glucuronide; AEC, anhydroecgonine; AEME, anhydroecgonine methyl ester; APAP, acetaminophen (paracetamol); APAP-O-Glu, acetaminophen-O-glucuronide; APAP sulfate, acetaminophen sulfate; BZE, benzoylecgonine; BZE-O-Glu, benzoylecgonine-O-glucuronide; CEC, cinnamoylecgonine; CCOC, cinnamoylcocaine; CE, cocaethylene; COC, cocaine; diOH-COC, dihydroxycocaine; DXM, dextromethorphan; DXO, dextrorphan; DXO-O-Glu, dextrorphan-O-glucuronide; EC, ecgonine; EME, ecgonine methyl ester; LEV, levamisole; MDZ, midazolam; NBZE, norbenzoylecgonine; NCOC, norcocaine; OH-BZE (I), hydroxybenzoylecgonine (isomer I); OH-BZE (II), hydroxybenzoylecgonine (isomer II); OH-BZE (III), hydroxybenzoylecgonine (isomer III); OH-COC (I), hydroxycocaine (isomer I); OH-COC (II), hydroxycocaine (isomer II); OH-COC (III), hydroxycocaine (isomer III); OH-MDZ-O-Glu, hydroxymidazolam-O-glucuronide; OH-NBZE, hydroxynorbenzoylecgonine; PHE, phenacetin.

ID	Matrix	Seed Node	Putative Assignment	Elemental Composition	ΔFormula (vs Parent)	Linked to	Direction(s)	MSn Score (%)	Coverage (%)	Matched Fragments	Confidence Level
1	Urine	COC	COC	C17 H21 N O4	-	OH-COC (isomer II)	Rev	77	76/79	25/26	1
2	Urine	COC	BZE	C16 H19 N O4	-(C H2)	OH-BZE (isomer I)	Rev	71	50/92	14/14	1
3	Urine	COC	NBZE	C15 H17 N O4	-(C2 H4)	NCOC	Fwd	73	47/100	4/4	2a
4	Urine	COC	OH-BZE (isomer I)	C16 H19 N O5	-(C H2) +(O)	COC seed	Fwd	46	71/20	5/5	2b
5	Urine	COC	OH-BZE (isomer II)	C16 H19 N O5	-(C H2) +(O)	COC seed	Fwd	56	86/26	6/6	2b
6	Urine	COC	OH-BZE (isomer III)	C16 H19 N O5	-(C H2) +(O)	OH-COC (isomer I)	Fwd	60	42/79	11/11	2b
7	Urine	COC	OH-NBZE	C15 H17 N O5	-(C2 H4) +(O)	OH-BZE (isomer II)	Fwd	70	48/92	11/11	2b
8	Urine	COC	NCOC	C16 H19 N O4	-(C H2)	COC seed	Fwd	40	57/23	4/7	2a

9	Urine	COC	OH-COC (isomer I)	C17 H21 N O5	+(O)	COC seed	Fwd	45	71/19	5/5	2b
10	Urine	COC	OH-COC (isomer II)	C17 H21 N O5	+(O)	COC seed	Fwd	43	71/15	5/5	2b
11	Urine	COC	OH-COC (isomer III)	C17 H21 N O5	+(O)	OH-BZE (isomer II)	Rev	58	46/70	16/16	2b
12	Urine	COC	diOH-COC	C17 H21 N O6	+(O2)	COC seed	Fwd	55	71/38	5/5	3
13	Urine	COC	CEC	C18 H21 N O4	+(C)	COC seed	Fwd	50	71/29	5/6	2b
14	Urine	COC	EME	C10 H17 N O3	-(C7 H4 O)	COC seed	Fwd	39	71/7	5/5	2a
15	Urine	COC	EC	C9 H15 N O3	-(C8 H6 O)	EME	Fwd	87	82/93	61/74	2a
16	Urine	COC	AEC	C9 H13 N O2	-(C8 H8 O2)	EME	Fwd	63	55/71	41/42	2a
17	Urine	COC	AEME	C10 H15 N O2	-(C7 H10 O2)	EME	Fwd	57	36/77	27/27	2a
18	Urine	COC	CCOC	C19 H23 N O4	+(C2 H2)	COC seed	Fwd	49	71/26	5/6	2a
19	Urine	COC	BZE-O-Glu	C22 H27 N O11	+(C5 H6 O7)	OH-BZE (isomer II)	Fwd	23	17/28	4/5	3
20	Urine	COC	CE	C18 H23 N O4	+(C H2)	COC seed	Fwd	61	71/50	5/5	2a
21	Urine	DXM	DXM	C18 H25 N O	-	DXM seed; 3-OH-morphinan	Fwd	83; 36	83/83; 44/28	15/15; 4/5	2a
22	Urine	DXM	DXO	C17 H23 N O	-(C H2)	DXM seed; DXM	Fwd	67	67/67	12/12	2a
23	Urine	DXM	3-methoxymorphinan	C17 H23 N O	-(C H2)	DXM seed; DXM	Fwd	60	33/86	6/6	2b
24	Urine	DXM	3-OH-morphinan	C16 H21 N O	-(C2 H4)	DXM seed; DXO; 3-methoxymorphinan	Fwd	42; 56; 76	28/56; 33/78; 86/67	5/5; 6/7; 6/6	2b
25	Urine	DXM	DXO-O-Glu	C23 H31 N O7	+(C5 H6 O6)	DXO; 3-methoxymorphinan; 3-OH-morphinan-O-Glu	Fwd	53; 57; 83	6-14-67/100	1/2; 2/2	2b
26	Urine	DXM	3-OH-morphinan-O-Glu	C22 H29 N O7	+(C4 H4 O6)	3-OH-morphinan; 3-methoxymorphinan	Fwd	61; 48	22/100; 29/67	2/3; 2/2	2b
27	Urine	MDZ	MDZ	C18 H13 Cl F N3	-	MDZ seed; OH-MDZ-O-Glu	Fwd	50	40/60	2/3	1
28	Urine	MDZ	OH-MDZ-O-Glu	C24 H21 Cl F N3 O7	+(C6 H8 O7)	MDZ seed	Fwd	88	75/100	3/3	2b
29	Urine	APAP	APAP	C8 H9 N O2	-	APAP seed	Fwd	83	80/86	12/12	1
30	Urine	APAP	APAP-O-Glu	C14 H17 N O8	+(C6 H8 O6)	APAP seed; APAP	Fwd	69; 58	67/61; 33/83	10/10; 5/6	2b
31	Urine	APAP	APAP sulfate	C8 H9 N O5 S	+(O3 S)	APAP seed; APAP	Fwd	55; 63	53/56; 64/63	8/9; 9/10	2b
32	Urine	APAP	PHE	C10 H13 N O2	+(C2 H4)	APAP seed; APAP; APAP-O-Glu	Fwd	67; 68	33-36/100	5/7	2a
33	Urine	LEV	LEV	C11 H12 N2 S	-	LEV seed	Fwd	83	100/67	12/12	2a
